# Recruitment of TREX to the Transcription Machinery by Its Direct Binding to the Phospho-CTD of RNA Polymerase II

**DOI:** 10.1371/journal.pgen.1003914

**Published:** 2013-11-14

**Authors:** Dominik M. Meinel, Cornelia Burkert-Kautzsch, Anja Kieser, Eoghan O'Duibhir, Matthias Siebert, Andreas Mayer, Patrick Cramer, Johannes Söding, Frank C. P. Holstege, Katja Sträßer

**Affiliations:** 1 Gene Center and Munich Center for Integrated Protein Science CIPSM at the Department of Biochemistry of the Ludwig-Maximilians-University of Munich, Munich, Germany; 2 Molecular Cancer Research, University Medical Center Utrecht, Utrecht, the Netherlands; The University of North Carolina at Chapel Hill, United States of America

## Abstract

Messenger RNA (mRNA) synthesis and export are tightly linked, but the molecular mechanisms of this coupling are largely unknown. In *Saccharomyces cerevisiae*, the conserved TREX complex couples transcription to mRNA export and mediates mRNP formation. Here, we show that TREX is recruited to the transcription machinery by direct interaction of its subcomplex THO with the serine 2-serine 5 (S2/S5) diphosphorylated CTD of RNA polymerase II. S2 and/or tyrosine 1 (Y1) phosphorylation of the CTD is required for TREX occupancy in vivo, establishing a second interaction platform necessary for TREX recruitment in addition to RNA. Genome-wide analyses show that the occupancy of THO and the TREX components Sub2 and Yra1 increases from the 5′ to the 3′ end of the gene in accordance with the CTD S2 phosphorylation pattern. Importantly, in a mutant strain, in which TREX is recruited to genes but does not increase towards the 3′ end, the expression of long transcripts is specifically impaired. Thus, we show for the first time that a 5′-3′ increase of a protein complex is essential for correct expression of the genome. In summary, we provide insight into how the phospho-code of the CTD directs mRNP formation and export through TREX recruitment.

## Author Summary

Gene expression is a fundamental cellular process that translates the information stored in the DNA into proteins, the workhorses of the cell. Eukaryotic cells contain a nucleus, where the genetic information is stored and transcribed by RNA polymerase II into messenger (m)RNAs. These copies of the blueprint of life need to be exported to the cytoplasm for protein production. Interestingly, mRNA synthesis is coupled to nuclear mRNA export. The protein complex TREX mediates this coupling of transcription to mRNA export. To assess the recruitment mechanism of TREX to genes we analyzed the presence of TREX over the whole genome in budding yeast. We found that there is more TREX at the end than at the beginning of genes. TREX binds to a subunit of RNA polymerase II, phosphorylation of which increases over the gene mediating the increase in TREX. Importantly, this increase in TREX over genes is important for normal levels of long transcripts. Thus, we show for the first time that a gradual increase of a protein complex is important for correct expression of the genome. We propose that TREX functions to keep the mRNA in the vicinity of the transcription machinery for correct processing and mRNP formation.

## Introduction

Gene expression is a fundamental process of every living cell. In eukaryotes, RNA polymerase II (RNAPII) transcribes protein-coding genes to synthesize messenger RNA (mRNA). In addition to RNAPII, a plethora of transcription factors is needed for efficient and regulated transcription in vivo. Transcription initiation factors recruit RNAPII to the promoter, whereas transcription elongation factors ensure efficient passage of RNAPII through the transcribed region [1]–[3]. Termination factors are required at the 3′ end of the gene to end the synthesis of mRNA. The newly synthesized mRNA is processed, *i.e.* capped, spliced and polyadenylated, and packaged into a ribonucleoprotein (mRNP) before its nuclear export. Interestingly, these downstream processes occur co-transcriptionally and are intimately linked to each other and to transcription to ensure efficient mRNA biogenesis [4].

The transcription cycle and the co-transcriptional processing of the mRNA are coordinated by the differential phosphorylation of the C-terminal domain (CTD) of Rpb1, the largest subunit of RNAPII [5]. The CTD consists of heptad repeats with the consensus sequence YSPTSPS with 26 or 52 repeats in budding yeast or humans, respectively. It serves mainly as a recruitment platform for transcription and mRNA processing factors, whose association is largely regulated by phosphorylation of the CTD at positions Y1, S2, T4, S5 and S7 [6]. S5 phosphorylation of the CTD is high during transcription initiation, decreases rapidly during the early phase of elongation, and persists at a low level throughout the body of the gene [7]–[10]. Consistently, the capping complex is recruited via direct interaction with the S5 phosphorylated CTD [6]. S2 phosphorylation appears early during transcription elongation, increases during the elongation phase and drops shortly 3′ of the polyadenylation (polyA) site [7]–[9]. Transcription elongation, splicing, termination and polyadenylation factors interact with the S2 or S2/S5 (di)phosphorylated CTD [5]. In *Saccharomyces cerevisiae*, the pattern of Y1 phosphorylation (Y1P) resembles that of S2 phosphorylation (S2P), with the exception of an earlier decrease at the poly(A) site [11]. Y1P stimulates binding of the transcription elongation factor Spt6 and prevents recruitment of termination factors [11]. S7 phosphorylation appears early at the 5′ end of genes, persists at a lower level throughout the open reading frame (ORF) and is required for the transcription and correct processing of human snRNA genes [7], [9], [12], [13]. Phosphorylation of T4 increases in the 3′ region of genes subsequently to the increase in S2P and is required for transcription elongation and histone mRNA 3′end processing [14], [15]. Hence, the CTD plays a pivotal role in the coordination of transcription with downstream processes.

In addition to the CTD, a multitude of proteins and protein complexes link transcription to one or several downstream events. In *S. cerevisiae*, the conserved TREX complex couples transcription to mRNA export [16]–[20]. TREX consists of the heteropentameric subcomplex THO, comprised of Tho2, Hpr1, Mft1, Thp2 and Tex1, the mRNA export factors Sub2 and Yra1 and the mRNA-binding proteins Gbp2 and Hrb1 [16]. TREX is essential for efficient transcription elongation and links transcription to mRNA export by recruiting the mRNA exporter Mex67-Mtr2 to the mRNA [18]–[20]. Furthermore, TREX also functions in 3′ end processing through its subunit Yra1, which is recruited to the polyadenylation factor by its interaction with Pcf11 [21], [22]. In addition, TREX prevents hyper-recombination events associated with inefficient mRNP assembly and functions in transcription-coupled DNA repair (TCR) [23], [24]. Thus, TREX is important for a multitude of co-transcriptional processes.

The TREX components Sub2, Yra1, Gbp2 and Hrb1 are thought to be transferred to the mRNA during packaging of the mRNA into a ribonucleoparticle (mRNP) [25], [26]. Yra1 (Aly in metazoans) directly interacts with the conserved mRNA exporter Mex67-Mtr2 in yeast (TAP-p15 or NXT-NXF in metazoans) and functions as an adaptor protein between this heterodimer and mRNA [27], [28]. Mex67-Mtr2/TAP-p15 binds directly to the mRNA as well as nuclear pore proteins and mediates export of the mRNP through the nuclear pore complex [18]–[20]. In addition to Yra1, the THO component Hpr1 and the mRNA-binding proteins Nab2 and Npl3, both of which are also recruited to the mRNA co-transcriptionally, are thought to function as adaptor proteins for Mex67-Mtr2 [29]–[31]. However, the specific function of the different proteins serving as adaptors has remained elusive.

TREX is recruited to all protein-coding genes but seems to be especially important for the expression of long and GC-rich transcripts since these are less abundant in deletion mutants of TREX [23]. The downregulation of long transcripts in TREX deletion mutants is consistent with the finding that THO is necessary for RNAPII processivity [32]. In addition, it has been long known that TREX moves along the gene together with RNAPII [16], but the molecular basis of this interaction has remained enigmatic. The Prp19 complex (Prp19C) interacts with TREX and RNAPII and is important to ensure TREX occupancy at the gene, especially at the 3′-end [33], [34]. However, Prp19C is not responsible for the recruitment of TREX to genes at the 5′ end [33]. Furthermore, the TREX subunit Yra1 has been shown to interact directly with the phospho-CTD, but it is currently unknown whether this interaction is needed for TREX recruitment [35]. Thus, it remained an open question how TREX is recruited to genes and how it interacts with the transcription machinery.

Here, we show that the occupancy of THO, Sub2 and Yra1 at genes increases from the 5′ to the 3′ end of the ORF and with gene length using ChIP-chip. A ChIP-based assay with a reporter construct containing a self-cleaving ribozyme shows that recruitment of TREX is at least partially RNA-dependent, but its 5′ to 3′ increase cannot be explained by RNA length. Instead, increasing TREX occupancy is most likely mediated by direct interaction of its subcomplex THO with the S2/S5 diphosphorylated CTD of RNAPII. Consistently, phosphorylation of the CTD on S2 is necessary for TREX recruitment in vivo. In contrast, THO recruitment is independent of Yra1's CTD-binding domain. Importantly, the 5′-3′ increase in TREX occupancy is crucial for the correct expression of long genes. This suggests that the CTD phospho-code dictates mRNP assembly and export through recruitment of TREX.

## Results

### TREX Recruitment Increases with Gene Length

The Mex67-Mtr2 heterodimer is recruited to mRNAs co-transcriptionally via association with multiple, distinct mRNA-binding proteins [29]–[31]. In *S. cerevisiae*, several proteins have been proposed to function as Mex67-Mtr2 adaptor proteins to the mRNA. This includes the TREX complex members Yra1 and Hpr1 as well as the mRNA-binding proteins Nab2 and Npl3. To assess whether these mRNA adaptors are differentially recruited to genes we assessed the genome-wide occupancy of individual TREX components, Nab2 and Npl3 by using high density tiling arrays for the analysis of chromatin immunoprecipitation (ChIP) experiments. TREX, Nab2 and Npl3 are recruited to all actively transcribed protein-coding genes with a slight preference of Npl3 for intron-containing genes and of Yra1 for intron-less genes (data not shown). This is consistent with previous data showing that Hpr1 and Sub2 are recruited to all ORFs [23]. TREX, Nab2 and Npl3 are also recruited to RNAPII-transcribed sn- and snoRNA genes, but at a lower level (Figure S1). This is consistent with the recent finding that in fission yeast THO is recruited to snoRNA genes, negatively regulating their expression [36]. Taken together, however, there is no marked difference in the recruitment of different mRNA export adaptors.

**Figure 1:**
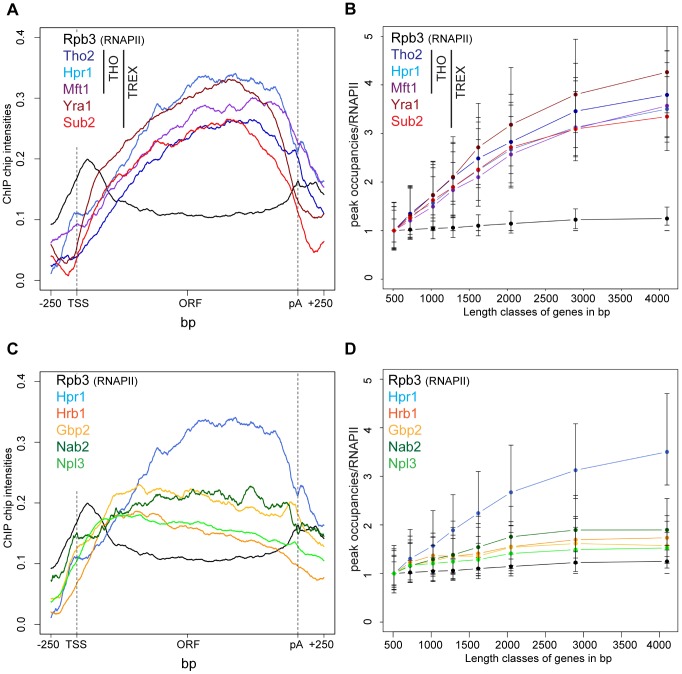
Recruitment of TREX increases from the 5′ to the 3′ end of the gene and with gene length. (A) The occupancy of the TREX components increases from 5′ to 3′. Meta gene occupancy profiles of the TREX components Tho2, Hpr1, Mft1, Sub2 and Yra1. The meta gene occupancy profile of Thp2 is similar (data not shown). For comparison, the meta gene occupancy profile of RNAPII (Rpb3) is shown. (B) The peak occupancies of TREX components increase with gene length. Genes were subdivided according to the indicated length classes and the TREX occupancy for each gene in each class was normalized to RNAPII occupancy. Length classes are: A (512–723 bp), B (724–1023 bp), C (1024–1286 bp), D (1287–1617 bp), E (1618–2047 bp), F (2048–2895 bp), G (2896–4095 bp), H (4096–5793 bp). (C) Meta gene occupancy profiles of the TREX components Gbp2 and Hrb1 and the mRNA-binding proteins Nab2 und Npl3. The occupancy of these mRNA-binding proteins decreases slightly towards the 3′ end of the gene. Gene occupancy was measured with both N- and C-terminally tagged for Gbp2 to ensure that the resulting profiles were not due to epitope tagging (data not shown). For Npl3, the occupancy of TAP-Npl3 was determined since Npl3-TAP mislocalizes to the cytoplasm. The meta gene occupancy profiles of RNAPII (Rpb3) and the TREX component Hpr1 are shown for comparison. (D) Occupancy of mRNA-binding proteins increases only slightly with gene length. Graph as in (B).

TREX travels along the gene together with RNA polymerase II [16] but the molecular basis for this interaction has remained elusive. In order to gain insight into the recruitment mechanism, we calculated meta gene occupancy profiles for each protein. To do this, the average nucleotide occupancy for each analyzed protein was plotted for the top 50% of the most highly transcribed genes (1,538–2,895 bp in length) after gene length normalization (Figure 1A). THO components, Sub2 and Yra1 appear at the transcription start site and their occupancies increase steadily from the 5′ to the 3′ end of genes (Figure 1A). Consistent with a function in mRNA export, the occupancy levels of all proteins drop at the polyA site and before the termination site (Figure 1A). These results are in accordance with genome-wide data published recently by Aguilera and coworkers for Hpr1 and Sub2 and data by the Rosbash lab for Yra1, Sub2 and Hpr1 recruitment to selected genes [23], [26]. According to the meta profiles, Sub2 and Yra1 dissociate from the transcription machinery slightly before the THO complex (Figure 1A). This might be due to a transfer of Sub2 and Yra1 to the mRNA (also see discussion). The increase of TREX components from 5′ to 3′ is striking since the occupancy of *bona fide* transcription elongation factors, such as Spt5, Spt6, Spt16, Bur1 and Paf1, does not increase over the length of the ORF (Figure S2). Thus, TREX might be the only transcription elongation complex whose occupancy increases with gene length.

The 5′ to 3′ increase in TREX occupancy could either lead to a maximal occupancy independent of gene length or to a higher maximal occupancy for longer genes in case the occupancy constantly increases until the 3′ end of the gene. In order to distinguish between these two possibilities, genes were subdivided into eight length classes between 500 bp and 5000 bp. The peak occupancy of TREX components at each gene within one length class was normalized to the occupancy of RNAPII (Rpb3) to correct for transcription activity, and the average normalized occupancy of TREX components was plotted for each length class (Figure 1B). As expected for the observed 5′ to 3′ increase, the average occupancy of all factors increases with gene length, *i.e.*, the longer a gene, the higher TREX occupancy. For genes shorter than 1500 bp this increase in TREX occupancy is roughly linear and lower for genes longer than 1500 bp. The 5′ to 3′ increase in TREX occupancy is also evident in meta gene occupancy profiles calculated for different length classes; the maximal occupancy of Tho2, Hpr1, Mft1, Sub2 and Yra1 increases with length of the gene class (Figure S3I, K, L, M, N). This increase in TREX occupancy with gene length is not caused by antisense transcription as the same increase is observed when genes containing CUTs or SUTs are omitted from the calculation (Figure S4). Taken together, the occupancy of THO, Sub2 and Yra1 increases from the 5′ to 3′ end of genes.

In contrast, the occupancy of TREX components Gbp2 and Hrb1 decreases slightly from 5′ to 3′ (Figure 1C) and increases only slightly with gene length (Figure 1D).This is also evident in the meta gene occupancy profiles of Gbp2 and Hrb1 for different length classes (Figure S3O,P). Interestingly, the occupancy of Nab2 and Npl3, two other mRNA-binding proteins in yeast important for mRNA export, is also constant over the ORF and increases only slightly with gene length (Figures 1D and S3Q,R). This difference in distribution over the length of the gene is also reflected by the correlations of the peak occupancies between the different proteins. For example, the THO subunits, Yra1 and Sub2 correlate highly with each other, whereas Gbp2 and Hrb1 correlate well with Nab2 and Npl3 and with general transcription elongation factors (Figure S5). In a study examining mRNP composition and structure it was suggested that the amount of Nab2 increases with RNA length [37], a finding that may apply to other mRNA-binding proteins present in the mRNP. Thus, Nab2, and other mRNA binding proteins such as Gbp2, Hrb1 and Npl3, may be removed from chromatin by transfer to the nascent mRNA. Regardless, this shows that the occupancy of a core TREX complex consisting of THO, Sub2 and Yra1 increases from 5′ to 3′ of the gene (also see Discussion).

### TREX Recruitment Is RNA-Dependent, but the 5′ to 3′ Increase in TREX Occupancy Is Not Caused by RNA Length

**Figure 2:**
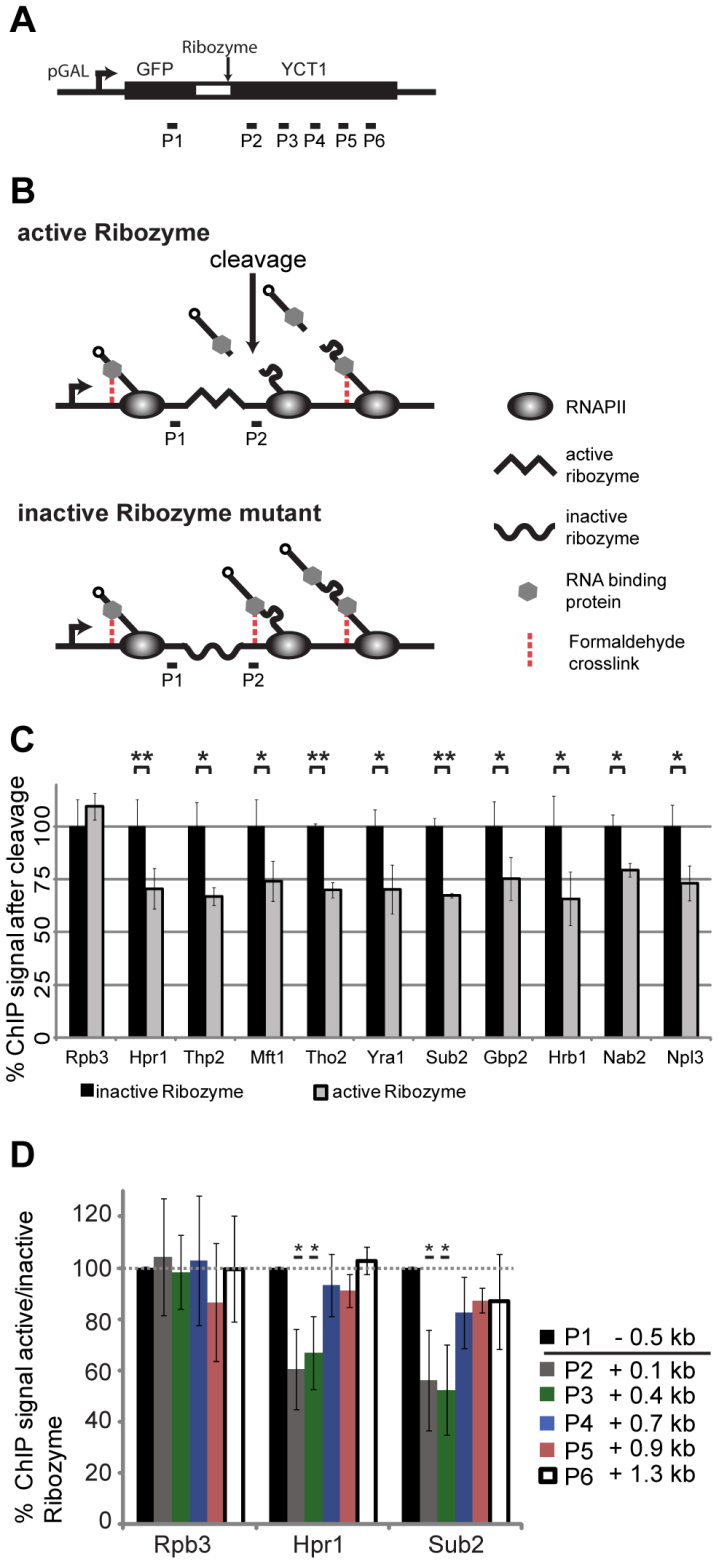
RNA is necessary for recruitment of TREX, Nab2 and Npl3 to chromatin, but not the cause for the 5′ to 3′ increase in TREX occupancy. (A) Scheme of the ribozyme containing reporter used to assess the dependence of TREX, Nab2 and Npl3 occupancy on RNA. A sequence coding for the *GAL1* promoter, GFP and the hepatitis δ ribozyme (wt or inactive mutant) was inserted 5′ of the nonessential *YCT1* gene. (B) Scheme of the ribozyme assay. Proteins tethered to chromatin by the mRNA are no longer chromatin-associated following cotranscriptional self-cleavage of the mRNA at the ribozyme sequence (upper panel, middle picture). The occupancy of each protein was compared to its occupancy at the same genomic position but a longer nascent mRNA attached by using a reporter construct with an inactive ribozyme (through mutation of one base pair) (lower panel, middle picture). The occupancy after the cleavage (P2) was normalized to the signal before the cleavage site (P1). Further downstream, when the RNA is long enough, the cleavage event will not influence the occupancy of RNA-binding proteins any more (both panels, right pictures). (C) Recruitment of TREX, Nab2 and Npl3 is dependent on RNA. For each protein the ChIP signal 3′ of the cleavage site (P2 in B) was normalized to the signal before the cleavage site (P1 in B) and set to 100% for the inactive ribozyme construct (black bars). The ratio of P2/P1 for the active ribozyme was calculated relative to the inactive ribozyme (grey bars). Whereas the signal for RNAPII (Rpb3) is unaffected by cleavage of the RNA, the signals for all TREX components, Nab2 and Npl3 drops to about 70% indicating a (partially) RNA-dependent recruitment of these mRNA-binding proteins. Results of at least 3 independent experiments are shown (mean +/− SD; **: p<0.01; *: p<0.05). (D) The 5′ to 3′ increase in TREX occupancy is independent of RNA length. ChIP signals for the TREX components Hpr1 and Sub2 and the RNAPII subunit Rpb3 before (P1) and at different genomic positions after the ribozyme cleavage site (P2–P6) were normalized to the signals in the inactive ribozyme mutant, which has an uncut and thus longer nascent transcript. This relative ChIP signal at P1 set to 100%. Recruitment of RNAPII (Rpb3) is independent of RNA cleavage. Between 400 and 700 bp after the cleavage site the occupancy of Hpr1 and Sub2 becomes independent of the cleavage. Results of at least 3 independent experiments are shown (mean +/− SD; **: p<0.01; *: p<0.05).

The 5′ to 3′ increase in TREX recruitment may be explained by interaction with the mRNA and/or the C-terminal domain (CTD) of Rpb1, the largest subunit of RNA polymerase II (RNAPII). First, we assessed whether the increased association of TREX towards the 3′ end of genes is caused by interaction with the growing mRNA. In order to assess the occupancy of a chosen protein dependent on the length of the RNA, it is necessary to cut the mRNA at a specific position to shorten the nascent RNA to a defined length in relation to which TREX occupancy can be measured. To do this, we established a hepatitis δ ribozyme based ChIP assay in *S. cerevisiae* according to [38] (Figure 2A). As the mRNA is synthesized, the internal ribozyme sequence folds into an enzymatically active RNA and initiates co-transcriptional self cleavage. This cleavage event releases the 5′ portion of the nascent mRNA and any proteins bound to it from chromatin, while RNAPII and the 3′ portion remain at the transcription site (Figure 2B). For each protein we measured its occupancy at a defined distance to the cleavage site. This occupancy was compared to the occupancy at the same position but with an uncleaved and thus longer RNA. As expected, the occupancy of RNAPII is not dependent on RNA (Figure 2C, Rpb3). In contrast, the occupancy of all TREX components, as well as Nab2 and Npl3, significantly decreases to about 70% 100 bp downstream of the ribozyme site (Figure 2C; P2). The efficiency of ribozyme cleavage in this context is not known. However, since protein occupancy decreases, a subset of transcripts must cleave soon after synthesis. This reduced occupancy indicates that the recruitment of TREX, Nab2 and Npl3 is at least partially dependent on RNA. It is of note that prior studies of the TREX components Sub2, Yra1 and Hpr1, demonstrated varying degrees of RNA-dependent interaction with chromatin [26]. Because our ribozyme cleavage assay affects each of these complex members equivalently, we suggest that discrepancies within this previous study are due to the use of RNAse digestion to assess RNA-dependent recruitment. More specifically, because nuclease digestion follows formaldehyde crosslinking steps, we speculate that this treatment may result in RNAse-resistant interactions that were RNA dependent in vivo. Our results suggest that the occupancy of all TREX components, as well as Nab2 and Npl3, is at least partially dependent on RNA at actively transcribed genes.

Importantly, though, we used this assay to determine, whether the 5′ to 3′ increase in the occupancy of TREX components is caused by the nascent RNA chain. With increasing RNA length proteins bound to the RNA are taken further and further away from the DNA template and might not be crosslinked to chromatin any more (Figure 2A, B). To test TREX occupancy dependent on nascent mRNA length, we analyzed the association of these factors at different downstream portions of the gene, distal to the ribozyme cleavage site (Figure 2A, P3–P6). Indeed, while the occupancy of Hpr1 and Sub2 is decreased 0.1 and 0.4 kb 3′ of the cleavage site compared to the inactive ribozyme sequence, *i.e.* at the same genomic position but with a longer nascent mRNA, it is unaffected 0.7 kb and further downstream of the cleavage site (Figure 2D). Thus, TREX occupancy is independent of RNA length once the nascent mRNA is longer than approximately 550 nt suggesting that the 5′ to 3′ increase in TREX occupancy observed over several kilobases is not caused by the growing mRNA chain.

### Y1 and/or S2 CTD Phosphorylation Is Essential for TREX Recruitment In Vivo

**Figure 3:**
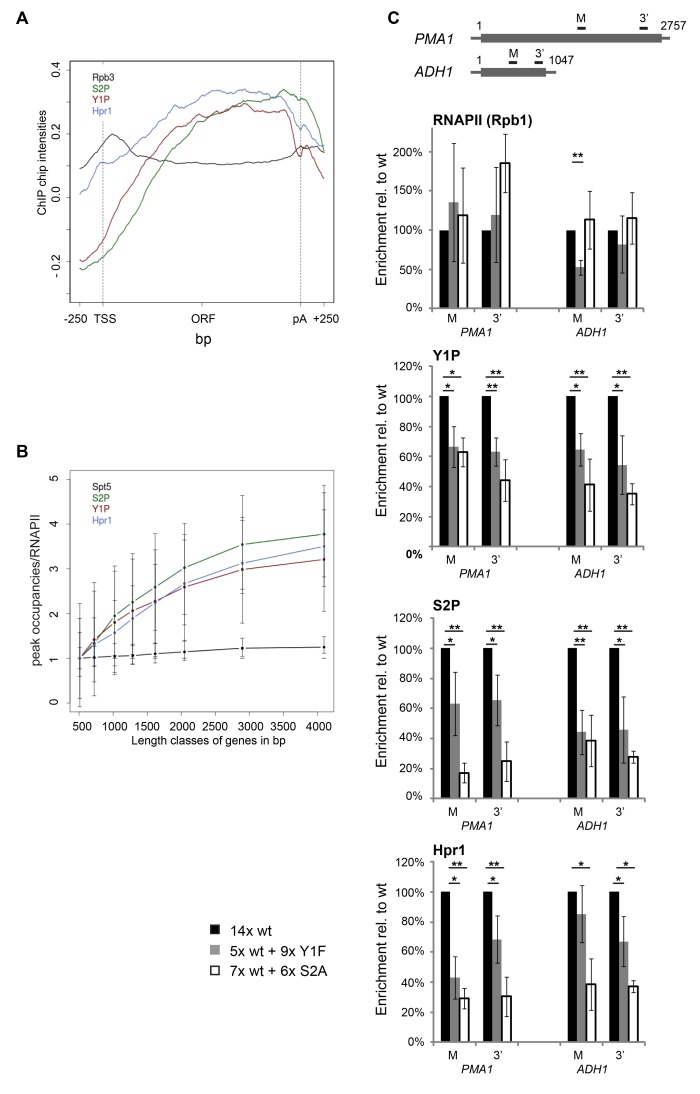
Y1 and/or S2 phosphorylation is essential for TREX recruitment in vivo. (A) The meta gene occupancy profiles of S2 and Y1 CTD phosphorylation parallels that of TREX occupancy. The meta gene occupancy profile of Hpr1 is shown for comparison. (B) The peak occupancies of Hpr1, S2P and Y1P increase with gene length. Graph as in Figure 1B. (C) Recruitment of RNAPII (Rpb1), Y1P, S2P and THO (Hpr1) to the *PMA1* (2757 nt) and *ADH1* genes (1047 nt). The uppermost panel depicts the *PMA1* and the *ADH1* and the position of the primer pairs. For *PMA1* the primer pair M amplifies nucleotides (nts) 1574–1651 and the primer pair 3′ amplifies nts 2484–2543; for *ADH1* primer pair M amplifies nts 408–476 and primer pair 3′ nts 916–966. The occupancies of Rpb1, Y1P, S2P and Hpr1 in the S2A (white bars) and the Y1F (grey bars) mutant strains were calculated relative to the occupancy in a strain with 14 wild-type CTD repeats (black bars). Results of at least 3 independent experiments are shown (mean +/− SD; **: p<0.01; *: p<0.05).

Another recruitment platform for TREX could be the CTD of RNAPII. The CTD is differentially phosphorylated during the transcription cycle and is well established to recruit a plethora of mRNA processing factors [5]. To assess this possibility, we compared recruitment of the TREX complex and RNA-binding proteins to the phosphorylation pattern of the CTD. Specifically, the meta gene occupancy profiles of Y1P and S2P are very similar to the ones of TREX with a biased distribution towards the 3′ end (Figure 3A). However, Y1P occupancy drops at the polyA site as does TREX occupancy, whereas S2P levels drop slightly downstream of the polyA site (Figure 3A). In addition, the peak occupancies of Y1P and S2P increase with gene length similar to TREX (Figure 3B). To test whether TREX occupancy depends on Y1 or S2 phosphorylation we used an S2A mutant with nine wild-type (wt) and six S2A repeats [39] and engineered an Y1F mutant carrying five wt and nine Y1F repeats. The remaining wild type repeats are necessary for survival since mutation of all S2 or Y1 residues is lethal [39]. A CTD truncated to 14 repeats served as wild-type control [39]. Interestingly, the S2A mutation leads to a decrease in Y1 phosphorylation and vice versa (Figure 3C, S2A and Y1P). This is not due to decreased RNAPII association, as the occupancy of RNAPII (Rpb1) is largely unaffected in both mutants at the *PMA1* and the *ADH1* gene (Figure 3C, RNAPII). This suggests that Y1 and S2 phosphorylation are interdependent, although we cannot exclude that our results are a reflection of epitope masking.

Importantly, the occupancy of Hpr1, and likely the whole THO complex, is also decreased in the Y1F and the S2A mutant showing that recruitment of THO is dependent on proper Y1 and/or S2 phosphorylation in vivo (Figure 3C). Consistently, occupancy of the TREX subunits Yra1 and Sub2 is impaired in the S2A mutant (Figure S6). S2 rather than Y1 phosphorylation is probably essential for TREX recruitment in vivo since sn/snoRNA genes are low in TREX occupancy and S2P but high in Y1P (Figure S1). However, the levels of Y1 and S2 phosphorylation most likely decrease in both CTD mutants, making it impossible to determine unambiguously which one of the two phosphorylation events is necessary for TREX occupancy in vivo.

### THO Is Recruited to the Transcription Machinery Independently of Yra1

**Figure 4:**
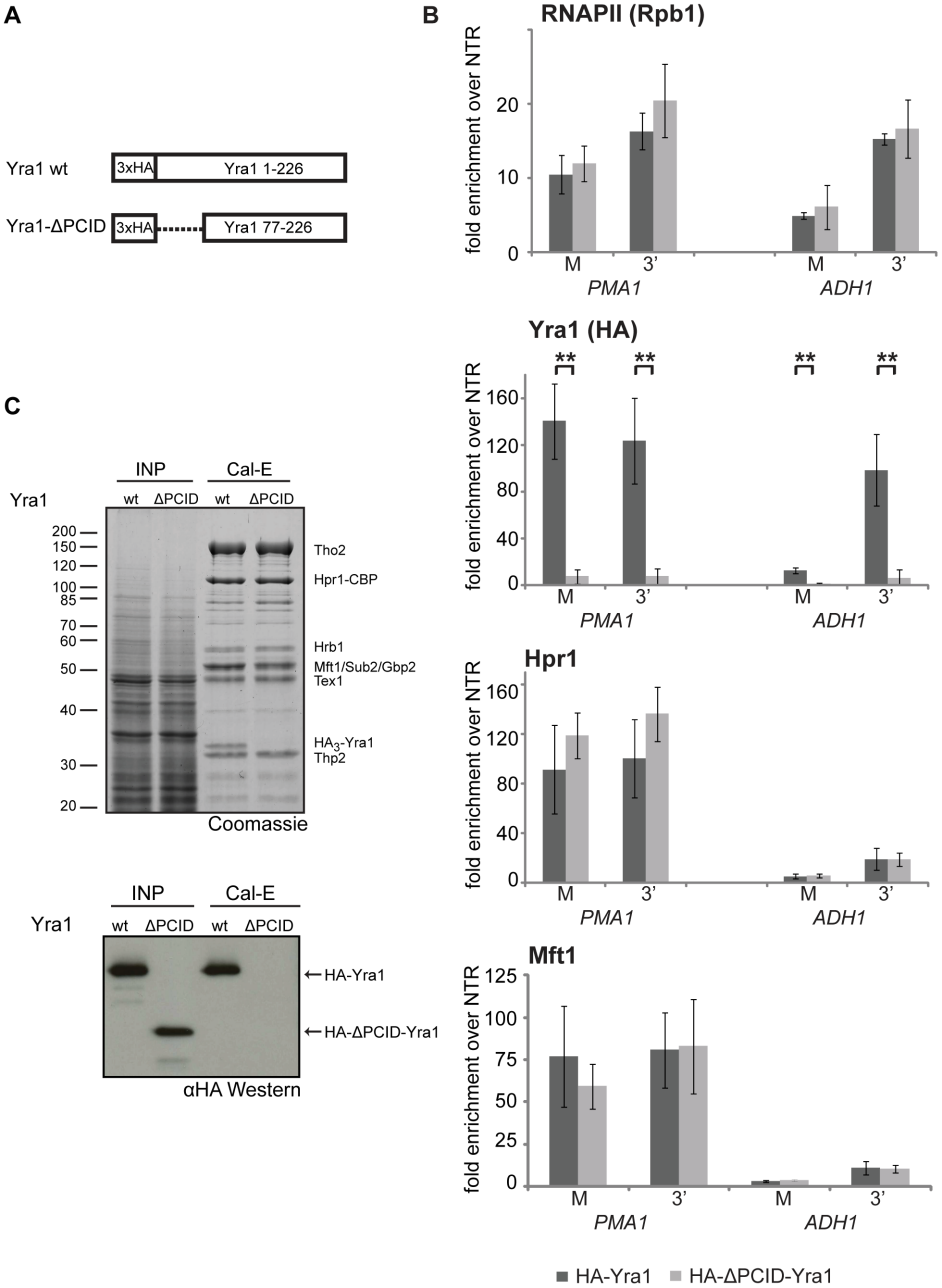
THO occupancy is independent of Yra1. (A) Schematic of the Yra1 wild-type and the ΔPCID mutant proteins. In the ΔPCID mutant the N-terminal 76 amino acids are absent, including the nuclear localization signal (NLS). (B) Deletion of the PCID of Yra1 leads to loss of Yra1 from the gene, but does not affect THO recruitment. The occupancies of RNAPII (Rpb1), Yra1 and THO (Hpr1, Mft1) at *PMA1* and *ADH1* in *YRA1* wild-type (dark grey bars) and *yra1-ΔPCID* cells (light grey bars) are shown. The positions of the primer pairs M and 3′ at the *PMA1* and the *ADH1* gene are as in Figure 3C. Results of at least 3 independent experiments are shown (mean +/− SD; **: p<0.01; *: p<0.05). (C) The PCID of Yra1 is essential for incorporation of Yra1 into TREX in vivo. TREX was purified from an *HPR1-TAP* strain expressing wild-type Yra1 (wt) or ΔPCID-Yra1 (ΔPCID). Lysates (INP) and calmodulin eluates (Cal-E) were stained with Coomassie (upper panel) and HA-tagged Yra1 was detected by Western blotting against HA (lower panel).

It has been shown recently that Yra1 binds to the S2/S5 diphosphorylated CTD in vitro and that deletion of the N-terminal 76 amino acids of Yra1 abrogates this interaction as well as recruitment of Yra1 to genes [35]. Thus, this N-terminal domain of Yra1 was named PCID for phospho-CTD interaction domain. In addition, the PCID also contains the NLS of Yra1 and is thus necessary for efficient nuclear localization of Yra1 [35]. However, it remained unclear whether this domain is also responsible for TREX recruitment to genes. In order to test whether the PCID of Yra1 is required for recruitment of TREX components in vivo, we assessed Yra1, Hpr1 and Mft1 occupancy in the *yra1-ΔPCID* mutant (Figure 4A). As shown before, Yra1 occupancy is greatly decreased in the *yra1-ΔPCID* mutant whereas RNAPII occupancy is not affected (Figure 4B, RNAPII and Yra1, and [35]). In contrast to Yra1, THO recruitment is not affected in the absence of Yra1 (Figure 4B, Hpr1 and Mft1). Thus, recruitment of THO is independent of Yra1.

Since recruitment of THO is not dependent on Yra1, we asked whether the interaction of Yra1 with THO could be impaired by deletion of the PCID. Full-length Yra1 copurified with TREX whereas yra1-ΔPCID did not (Figure 4C). The lack of yra1-ΔPCID incorporation into the TREX complex might be due to three reasons: 1. the PCID being necessary for the interaction of Yra1 with the other TREX components, 2. the mislocalization of yra1-ΔPCID to the cytoplasm, and/or 3. an impaired interaction of Yra1-ΔPCID with Pcf11, which is needed for recruitment of Yra1 [21] and from which Yra1 – after recruitment – could be transferred to THO. Important in this context, THO is recruited to the transcription machinery independently of Yra1.

### THO Binds Directly to the S2/S5 Diphosphorylated CTD

**Figure 5:**
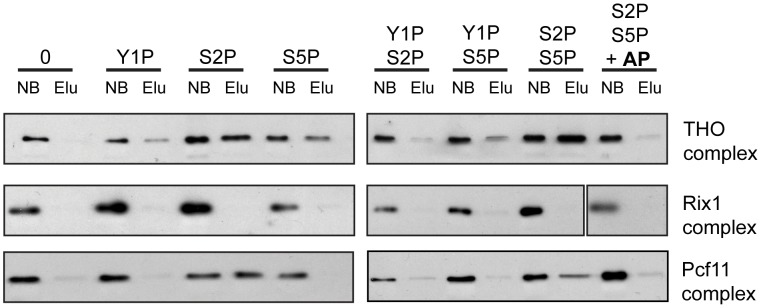
THO binds directly to the S2/S5 diphosphorylated CTD. Pulldown experiments were performed with immobilized CTD peptides that were not phosphorylated (0), mono-phosphorylated on tyrosine 1 (Y1P), serine 2 (S2P) or serine 5 (S5P), diphosphorylated on Y1/S2 (Y1PS2P), Y1/S5 (Y1PS5P) or S2/S5 (S2PS5P) or S2PS5P dephosphorylated by treatment with alkaline phosphatase (S2PS5P+AP). The THO complex binds to CTD peptides phosphorylated on S2 (S2P) and S5 (S5P) and more strongly to the S2/S5 diphosphorylated CTD (S2PS5P). Binding of THO to the CTD is dependent on S2/S5 diphosphorylation since treatment with alkaline phosphatase (AP) of the S2/S5 diphosphorylated CTD peptide abrogates binding of THO (S2PS5P+AP). The unrelated Rix1 complex served as negative control. Pcf11 was used as a positive control for association with the S2 and S2/S5 (di)phosphorylated CTD. The TAP-tagged protein of each complex was detected by Western blotting against CBP (Hpr1 for THO, Rix1 for the Rix1 complex and Pcf11 for the Pcf11 complex). A representative experiment is shown.

In order to assess whether recruitment of THO is mediated by direct binding of THO to the phosphorylated CTD and which phosphorylation event is necessary for this interaction, we performed pulldown experiments. CTD peptides that were either unphosphorylated, monophosphorylated on Y1, S2 or S5 or diphosphorylated on Y1 and S2, Y1 and S5 or S2 and S5 were immobilized on beads and incubated with the endogenous THO complex purified from yeast under high salt conditions. This purification method yields a pure THO complex composed of Tho2, Hpr1, Mft1, Thp2 and Tex1 but lacking Sub2, Yra1, Gbp2 and Hrb1 (Figure S7). The unrelated Rix1 complex, which is required for processing of ITS2 sequences from the 35S pre-rRNA, served as negative control [40], [41]. Pcf11 served as a positive control since this 3′end processing factor binds to the S2 phosphorylated CTD [42], . THO binds to the S2 and the S5 monophosphorylated CTD and exhibits the strongest interaction with the S2/S5 diphosphorylated CTD (Figure 5, upper panel). In contrast, THO did not bind to the Y1 phosphorylated CTD peptides (Figure 5). When the S2/S5 diphosphorylated CTD peptides were treated with alkaline phosphatase (AP) the interaction between THO and the CTD was abrogated, showing that the interaction is indeed phosphorylation dependent (Figure 5, upper panel). Thus, THO associates directly with the S2/S5 diphosphorylated CTD. This is consistent with the requirement for S2 phosphorylation for TREX occupancy in vivo (Figure 3) and the increase in occupancy towards the 3′ end (Figure 1). Since S2P increases from 5′ to 3′ and with the length of the gene while S5P peaks at the 5′ end and persists at a basal level throughout the gene (Figure S3C,D and [44]) the binding of THO to the S2/S5 diphosphorylated CTD is most likely the molecular basis for the 5′ to 3′ increase of THO, Sub2 and Yra1.

### The 5′-3′ Increase in TREX Occupancy Is Important for the Expression of Long Transcripts

**Figure 6:**
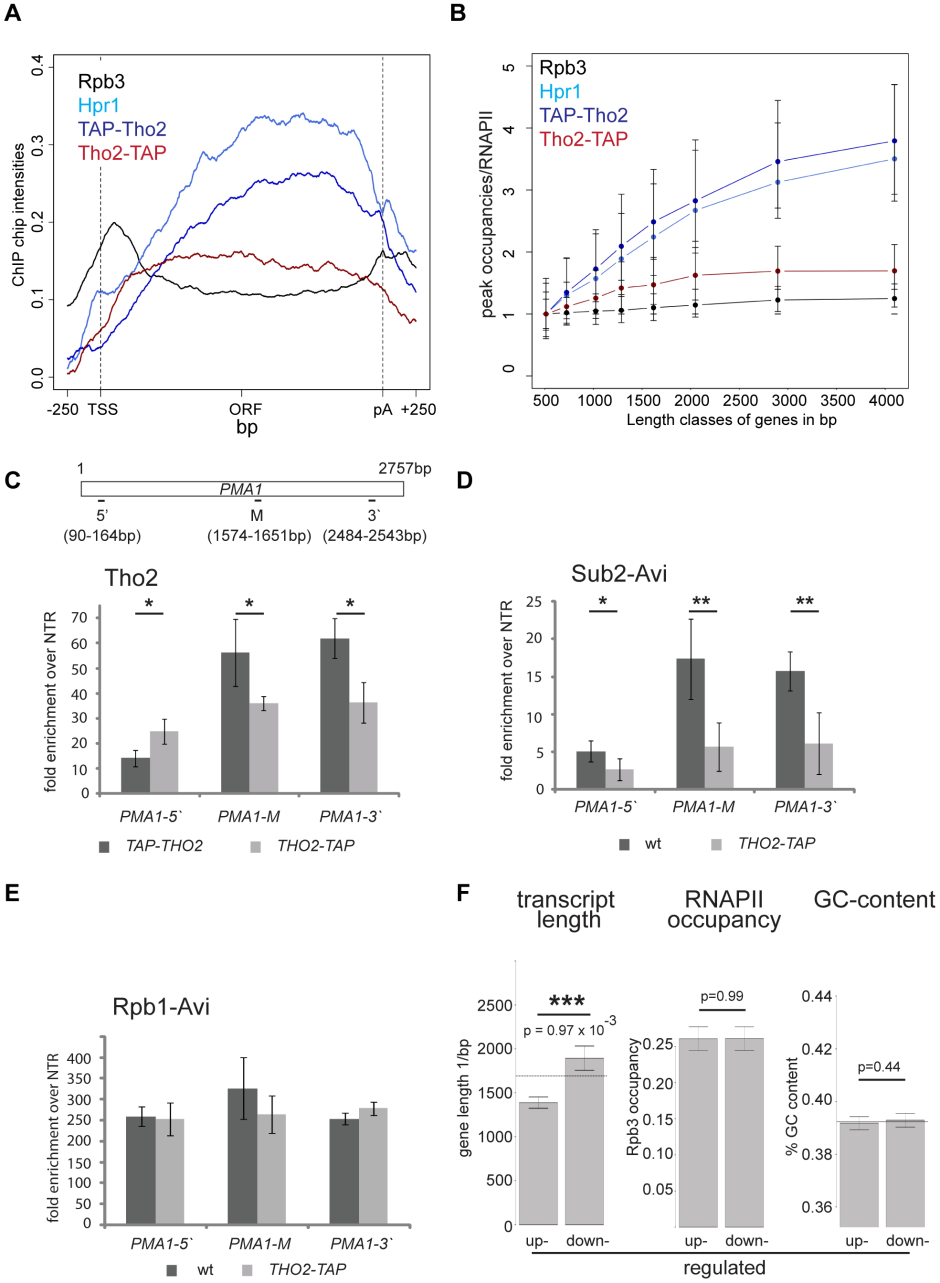
The increase in TREX occupancy is important for the expression of long transcripts. (A) Meta gene occupancy profiles of RNAPII (Rpb3), Hpr1, TAP-Tho2 and Tho2-TAP. Whereas TAP-Tho2 shows the 5′-3′ increase typical for THO/TREX components, Tho2-TAP is recruited to genes, but does not increase towards the 3′ end of the gene. The Y-intercept of Tho2-TAP was adjusted with −0.05 in order to superimpose Tho2-TAP and TAP-Tho2 at the transcription site for better visualization. (B) Occupancy of Tho2-TAP does not increase with gene length as other TREX components. Peak occupancy of Hpr1, TAP-Tho2 and Tho2-TAP in comparison to the *bona fide* transcription elongation factor Spt5. (C–E) TREX is recruited to the *PMA1* gene, but its occupancy does not increase in the *THO2-TAP* mutant. Occupancy of Tho2 (C), Sub2 (D) and Rpb1 (E) at the *PMA1* gene in the *TAP-THO2* and the *THO2-TAP* strain. Results of 3 independent experiments are shown (mean +/− SD; **: p<0.01; *: p<0.05). To assess the occupancy of Hpr1 and Sub2 in the presence of the TAP-Tag on Tho2, they were tagged with the Avi-tag. (F) Expression of long transcripts is downregulated in the *THO2-TAP* strain whereas highly transcribed and GC-rich transcripts are not affected. Microarray analysis reveals that transcripts upregulated in the *THO2-TAP* strain are shorter than the average of all transcripts, whereas downregulated transcripts are longer. The line indicates the average length or GC-content of all genes while the bars represent the average gene length, RNAPII occupancy or GC-content of up- or down-regulated genes, respectively. The error bars show the SEM and the p-value was calculated using the Wilcoxon rank sum test.

Importantly, we asked whether the 5′-3′ increase of TREX is physiologically relevant. Analysis of the transcriptomes of TREX knock-out mutants would make effects due to the lack of the whole protein indistinguishable from effects caused by the lack of the 5′-3′ increase. Thus, we exploited an allele of *THO2* encoding a C-terminally TAP-tagged Tho2 that fortuitously results in defective recruitment of TREX towards the 3′ end. In contrast to the N-terminally TAP-tagged Tho2, TAP-Tho2, the occupancy of Tho2-TAP neither increases from 5′ to 3′ nor with gene length genome-wide (Figure 6A,B). Since the signals obtained from genome-wide experiments are not quantitative, we determined the levels of Tho2-TAP and TAP-Tho2 recruitment by ChIP followed by quantitative RT-PCR for different regions of the *PMA1* gene. Importantly, Tho2-TAP and TAP-Tho2 are recruited to similar levels to the 5′ end of *PMA1* (Figure 6C). Next, we wanted to assess whether recruitment of the whole TREX complex is impaired similarly to Tho2-TAP. Since the protein A moiety of the TAP tag interferes with the use of any antibody, the other TREX components were tagged with the avidin epitope tag (Avi-tag). This bacterial biotin-acceptor peptide is biotinylated in cells expressing the corresponding biotin ligase BirA and can be immunoprecipitated with streptavidin beads [45]. Hpr1, Sub2 and Yra1 are recruited to similar levels to the 5′end of genes in wt and *THO2-TAP* cells, but do not increase towards the 3′ end in the *THO2-TAP* mutant (Figures 6D and S8A,B). Transcription by RNAPII is largely unaffected as judged by the fact that RNAPII occupancy does not change significantly in the *THO2-TAP* strain (Rpb1-Avi, Figure 6E). In addition, TAP-Tho2 and Tho2-TAP are assembled into the TREX complex (Figure S8C). Thus, the TREX complex is intact and recruited to the 5′ end of genes, but no longer shows 3′ end biased occupancy in the *THO2-TAP* strain.

In order to assess the physiological relevance of TREX's increasing occupancy, the transcriptomes of the *THO2-TAP* and a corresponding wild-type strain were analyzed for transcripts with differential expression. Importantly, the expression of long transcripts is decreased in the *THO2-TAP* strain (Figure 6F). In contrast, the expression of other gene classes, including highly expressed, highly transcribed, GC-rich, convergent and divergent genes, does not change when the 5′-3′ increase in TREX recruitment is impaired (Figures 6F and S9). In addition, the position of the first nucleosome or the promoter type does not influence expression in the *THO2-TAP* strain (Figure S9). Thus, the 5′ to 3′ increase in TREX occupancy is important for the expression of long transcripts. Interestingly, *THO2-TAP* is synthetically lethal with *yra1-ΔPCID*, *i.e.* when Yra1 is largely mislocalized to the cytoplasm and mRNA export compromised (Figure S10). This finding underlines the physiological importance of the 5′ to 3′ increase of TREX.

In summary, we identified a direct interaction of TREX with the S2/S5 diphosphorylated CTD of RNAPII that most likely mediates the 5′-3′ increase in TREX occupancy important for the expression of long genes. Thus, the differential phosphorylation of the CTD not only coordinates transcription and mRNA processing, but also couples transcription to mRNA export via TREX recruitment.

## Discussion

**Figure 7:**
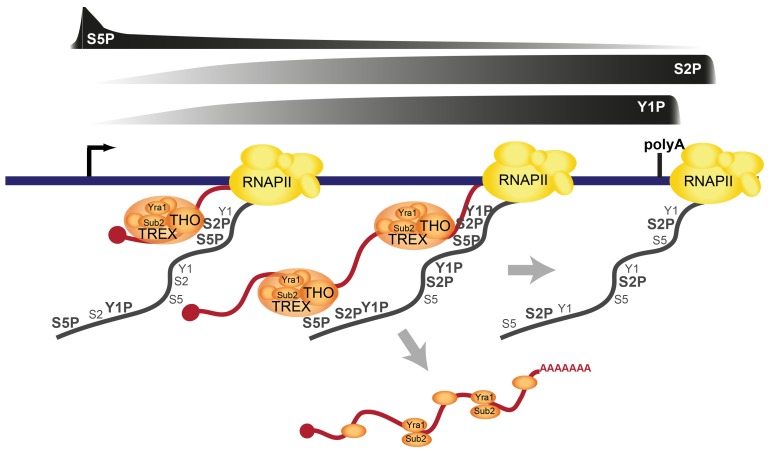
Model of TREX recruitment and dissociation. TREX interacts directly with the S2/S5 diphosphorylated CTD and RNA. Both interactions are important for recruitment of TREX to transcribed genes. The increasing S2 phosphorylation during transcription elongation leads to an increased occupancy of TREX towards the 3′ end of the gene. Importantly, this increase in TREX occupancy is crucial for the correct expression of long transcripts. As mRNA length increases, additional TREX complexes might be necessary to keep the nascent mRNA in the vicinity of the CTD, which recruits additional factors for mRNA processing and packaging prior to transcript release. In addition to TREX, multiple other proteins important for mRNA processing and packaging as well as the mRNA exporter Mex67-Mtr2 are recruited to the mRNA cotranscriptionally (not depicted). At the 3′ end of genes, TREX is dissociated from the transcription machinery and the chromatin. This may involve a single mechanism or multiple, biochemically distinct events. Sub2 and Yra1 most likely associate with the fully mature mRNP and leave the site of transcription. See text for details.

The TREX complex is essential for gene expression through its functions in transcription elongation, 3′end processing and mRNA export. It has been known for years that TREX is recruited to the transcription machinery. However, how TREX interacts with RNAPII has remained enigmatic. Here, we show that TREX binds directly to RNAPII through the direct interaction of its subcomplex THO with the S2/S5 diphosphorylated CTD of Rpb1 (Figure 7). THO is thus a new member of a small but growing class of protein complexes that bind to the S2/S5 double mark. Other S2/S5 diphosphorylated CTD binding proteins are Set2, which methylates histone H3 during transcription elongation, and Rco1, a subunit of the RPD1S complex, which deacetylates H3 and H4, preventing cryptic transcription [46]–[49]. TREX is recruited to genes early during transcription elongation and increases in occupancy as elongation proceeds. This increase in TREX occupancy is most likely mediated by the increase in S2 phosphorylation (Figures 3 and 5). The importance of S2 phosphorylation is consistent with the finding that Ctk1, the S2 kinase, physically and genetically interacts with TREX [25]. In addition to the CTD, RNA is necessary for TREX recruitment (Figure 2C). Interestingly, it was recently shown that the C-terminus of Tho2 interacts with nucleic acids and is necessary for occupancy of THO at transcribed genes [50]. Thus, the lack of the 5′-3′ increase in TREX occupancy in the Tho2-TAP mutant could be due to the fact that the C-terminal TAP tag interferes with nucleic acid binding, *i.e.* either of DNA or RNA. This is consistent with the dependence of TREX recruitment on RNA. However, the elongating RNA chain is not necessary for the increase in TREX occupancy along the gene (Figure 2D). Taken together, we propose the model that TREX is recruited to the transcription machinery by interaction of THO with RNA and the S2/S5 diphosphorylated CTD (Figure 7). The 5′ to 3′ increase in S2 phosphorylation mediates a corresponding increase in TREX occupancy (Figure 7).

Previously, we showed that the Prp19 splicing complex (Prp19C) is not necessary for initial recruitment of TREX to the 5′ end of genes, but rather ensures TREX occupancy along the gene unit [33]. This suggests that Prp19C functions to stabilize the interaction between TREX and the transcription machinery. Our studies above now complement this finding by demonstrating a direct interaction between TREX and the S2/S5 diphosphorylated CTD (see above).

Consistent with earlier observations [44] TREX leaves the gene at the polyadenylation site suggesting its dissociation from RNAPII before transcription termination (Figures 1 and 7). The dissociation of TREX could be brought about by the decrease in S2 phosphorylation at the polyadenylation site. However, TREX could also be dissociated by termination factors that bind to the CTD when Y1 phosphorylation decreases and polyadenylation factors are recruited [11], [44] (Figure 7). The latter scenario seems especially likely since the meta gene occupancy profiles of TREX components more closely resemble that of Y1P than S2P (Figure 3A) but THO does not bind to the Y1 phosphorylated CTD (Figure 5). In addition, TREX dissociation could be enhanced by loss of Prp19C function and/or cleavage of the mRNA during 3′ end formation. Thus, multiple processes may ensure the timely dissociation of the proteins necessary for mRNP formation (Figure 7). Sub2 and Yra1 might leave the transcription machinery as part of the mRNP (Figure 7), whereas THO might either bind to the mRNP or be directly recycled for a new round of transcription (not shown).

Although highly speculative, the interaction of THO with the phospho-CTD could be conserved in metazoans. A direct interaction of TREX with the transcription machinery might be the basis for the association of TREX with naturally intronless mRNAs via a sequence element termed CAR-E [51]. TREX could then recruit the splicing machinery to transcribed genes, consistent with the largely cotranscriptional splicing in higher eukaryotes. Interestingly in this context, a human transcription elongation factor, CA150, binds directly to the phospho-CTD and to the splicing factor SF1 repressing transcription elongation [52], [53]. Conversely, metazoan TREX could be recruited to the transcription machinery by the spliceosome. This seems especially likely if human TREX also interacts with Prp19C, which is recruited to the transcription machinery by direct interaction with the splicing factor U2AF65, which in turn interacts directly with the phospho-CTD [54]. In any event, it will be interesting to see whether TREX recruitment to the mRNA also increases towards the 3′-end of the gene in higher eukaryotes and, if so, whether TREX will be important for the expression of long transcripts.

Although the major components of TREX, THO, Sub2 and Yra1, show a 3′ biased distribution, Gbp2 and Hrb1, two members of the TREX complex, do not. The mRNA-binding proteins, Nab2 and Npl3, also show equal occupancy across both long and short genes. We hypothesize that these mRNA-binding proteins are transferred to the mRNA during transcription elongation and thus leave the transcription site. Interestingly, Npl3 – which is not a TREX component – also binds to the S2 phosphorylated CTD [55]. Therefore, mRNA-binding proteins may be recruited to the site of transcription by interacting either directly or indirectly, *i.e.* via THO, with the phosphorylated CTD. During transcription elongation these mRNA-binding proteins are then transferred from the CTD to the mRNA packaging it into an mRNP.

The occupancy of THO, Sub2 and Yra1 increases from the 5′ to the 3′ end of the gene. This makes this “core” TREX complex unique among the transcription elongation factors (Figure S2) as well as known S2P-CTD-interacting proteins (Figure S11). Importantly, this 5′-3′ increase is physiologically important. Exploiting a mutant of *THO2*, in which TREX is recruited to the gene but does not increase towards the 3′ end of the gene (Tho2-TAP), we show that the 5′-3′ increase in TREX occupancy is important for the correct expression of long genes. THO, Sub2 and Yra1 might be needed at higher levels towards the 3′ end of genes, to keep the nascent mRNA in the vicinity of the CTD (Figure 7). This could be necessary to ensure efficient and correct processing and packaging of the mRNA, which is consistent with the finding that a continuous transcript is needed for mRNA processing [38]. A fully extended CTD is approximately 700 Å long, which corresponds to the length of a 2.5 kb long linear mRNA. Since the median length of an mRNA in *S.c.* is 1.436 nucleotides [56], the CTD is in principle able to span the entire length of an average mRNA. However, it is unlikely that the CTD as well as the mRNA exist in a fully extended form in vivo. Thus, it remains to be elucidated, how mRNP formation is spatially organized. We propose that TREX promotes mRNP packaging through its bifunctional binding to the CTD and RNA by ensuring spatial proximity of the nascent mRNA to mRNP binding proteins, which are recruited to the CTD. In summary, we identify a direct interaction of TREX with the phospho-CTD as one molecular mechanism of TREX recruitment to transcribed genes (Figure 7). Thus, in addition to its many known functions the CTD code probably also coordinates transcription with mRNA export.

## Materials and Methods

### Strains and Plasmids

Yeast strains and plasmids are listed in Tables S1 and S2, respectively.

### ChIP and ChIP-chip Experiments

Chromatin immunoprecipitation (ChIP) assays were performed according to [57] and ChIP-chip experiments as in [7], [11]. ChIP-chip of Rpb3-TAP, TAP-Tho2, Tho2-TAP, Hpr1-TAP, Mft1-TAP, Gbp2-TAP, Hrb1-TAP, Nab2-TAP and TAP-Npl3 was performed using IgG coupled Sepharose beads (GE). Antibodies directed against the protein were used for Yra1 [16], Sub2 [16], Y1P [11] and S2P [12]. Details and differences are described in the Supplementary Material. The ChIP-chip data has been deposited in ArrayExpress (www.ebi.ac.uk/arrayexpress/), accession number E-MTAB-1400.

### Expression Profiling

Tho2-TAP and wild type cells were grown in SDC media, RNA was isolated and hybridized in dye-swap biological replicate to dual-channel 70-mer oligonucleotide arrays to obtain four measurements as previously described [58]. Up- or down-regulation of expression in the *THO2-TAP* strain was defined as a >1.7-fold change versus the average wild-type with a p-value of <0.05. The average length, GC-content, expression level, RNAPII occupancy, convergence, divergence, +1 and +2 nucleosome positioning of the up- and down-regulated genes was calculated and the statistical significance determined using the Wilcoxon rank sum test. SAGA and TFIID dominated genes were analyzed for expression changes versus all promoters using the Wilcoxon rank sum test. The microarray gene expression data has been deposited in ArrayExpress (www.ebi.ac.uk/arrayexpress/), accession number E-MTAB-1892.

### Tandem Affinity Purifications

Tandem affinity purifications (TAPs) were essentially done as described previously [16]. HA-tagged Yra1 was detected with an anti-HA antibody (Roche). Protein complexes used in the pulldown assays were purified from yeast until the TEV eluate and for THO and TREX followed by a second purification step using metal ion affinity chromatography. Details are given in the Supporting Information.

### CTD-Peptide Pulldown Assay

Pulldown assays were performed as described previously with following modifications [59]. For each pulldown assay 15 µl of Streptavidin coupled magnetic beads (Invitrogen) were washed three times with HS buffer (1 M NaCl, 25 mM Tris/HCl, pH 8.0, 5% Glycerol, 2.5 mM DTT, 0.025% NP-40, 0.1% BSA). Beads were resuspended in 100 µl HS buffer and incubated with 10 µg of each peptide for 2 h at 4°C. Peptides sequences are listed in Table S3. Peptides were ordered from PSL (Heidelberg) and PANAtecs (Tübingen). Beads were then washed once with HS buffer and two times with LS buffer (100 mM NaCl, 25 mM Tris/HCl, pH 8.0, 5% Glycerol, 2.5 mM DTT, 0.025% NP-40, 0.1% BSA). For alkaline phosphatase (AP) treatment samples were washed two times with 1× fast digestion buffer (Fermentas), incubated for 15 min at 37°C with 25 U FastAP (Fermentas), washed 2× with LS buffer and resuspended in 100 µl LS buffer. To test CTD binding equal amounts of the different protein complexes (typically 5–10 µl) were incubated with the CTD-coupled beads for 90 min at 4°C. The non-bound fraction was collected. After 4 washing steps with 500 µl LS buffer beads were resuspended in 1× gel-loading buffer to elute the bound protein complexes. Non-bound and bound protein complexes were detected with an anti-CBP antibody (Open Biosystems, CAB 1001) recognizing the remaining CBP-tag on the tagged proteins (Hpr1, Rix1 and Pcf11, respectively).

### Accession Codes

Raw and normalized data are available at ArrayExpress (www.ebi.ac.uk/arrayexpress/), accession numbers E-MTAB-1400 (ChIP-chip data) and E-MTAB-1892 (microarray gene expression data).

## Supporting Information

Figure S1 TREX is recruited to all RNAPII-transcribed genes. (A) Peak occupancies of the indicated proteins relative to RNAPII (Rpb3) on protein coding genes. The lower and upper borders of the boxes reflect the 25% and 75% quantiles, respectively, the black lines are the median values and the whiskers extend to the 1.5-fold inter quartile range. The red line gives the ratio 0, corresponding to no recruitment, and the dashed red line represents a ratio of 2. (B) Peak occupancies as in (A) but for sn/snoRNA genes.
(TIF)

Figure S2 The occupancy of *bona fide* transcription elongation factors does not increase during transcription elongation. (A) Meta gene occupancy profiles and (B) peak occupancy according to length classes for RNAPII (Rpb3) (only A), the transcription elongation factors Spt5 (only B), Spt6, Spt16, Bur1 and Paf1 and the TREX component Hpr1. The peak occupancies in (B) were normalized to the peak occupancy of RNAPII to correct for the rate of transcription.
(TIF)

Figure S3 TREX occupancy increases with gene length. (A–Q) Meta gene occupancy profiles of RNAPII (Rpb3) (A), the CTD phosphomarks Y1P (B), S2P (C) and S5P (D), the transcription elongation factors Spt5 (E), Spt6 (F), Spt16 (G) and Paf1 (H), the THO components Tho2 (I), the mis-recruited allele Tho2-TAP (J), Hpr1 (K) and Mft1 (L), the TREX components Sub2 (M), Yra1 (N), Gbp2 (O) and Hrb1 (P) and the mRNA-binding proteins Nab2 (Q) and Npl3 (R) were calculated for different gene classes. The gene classes were defined as in [7]: S (512–937 bp, 266 genes), M (938–1537 bp, 339 genes) and L (1538–2895 bp, 299 genes). The number of genes in each class is given in parentheses.
(TIF)

Figure S4 The 5′ to 3′ increase of TREX is independent of antisense transcription. The peak occupancy of TREX components Tho2, Hpr1, Mft1, Sub2 and Yra1 increases with gene length when genes containing a SUT or CUT 250 bp up- or downstream of the ORF were excluded from the calculation [60]. Calculations as for Figures 1B and S2.
(TIF)

Figure S5 Pearson correlation coefficients between TREX components, Nab2, Npl3 and general elongation factors. The peak occupancies (90^th^ percentile of each profile for each gene) of each protein were correlated for all protein coding genes. As expected, the general elongation factors Spt5, Spt6 and Elf1 correlate very highly with each other. Also RNAPII (Rpb3) and the phospho-CTD marks Y1P, S2P and S5P correlate very well with each other and with the general elongation factors. S2P strongly correlates with THO subunits, Yra1 and Sub2, and more weakly with general elongation factors. As expected, the THO subunits, Sub2 and Yra1 correlate highly with each other. Due to their lack of length dependency Gbp2 and Hrb1 correlate less with THO subunits, Sub2 and Yra1, but highly with Nab2 and Npl3.
(TIF)

Figure S6 S2 phosphorylation is essential for recruitment of Sub2 and Yra1. The occupancies of Sub2 and Yra1 in the S2A mutant strain (white bars) were calculated relative to the occupancy in a strain with 14 wild-type CTD repeats (black bars). Results of at least 3 independent experiments are shown (mean +/− SD; **: p<0.01; *: p<0.05).
(TIF)

Figure S7 Tandem affinity purification (TAP) of the THO complex used in the CTD pulldown experiments. A strain expressing C-terminally TAP-tagged Hpr1 and C-terminally His6-tagged Mft1 was purified by two steps using IgG and Ni affinity purification under low salt (100 mM NaCl) and high salt (1000 mM NaCl) conditions yielding the whole TREX complex or the THO complex consisting of Tho2, Hpr1, Mft1, Thp2 and Tex1, respectively. (A) Coomassie stain of eluates after Ni affinity purification. The identity of each protein was verified by mass spectrometry and is indicated to the right. (B) Yra1 is absent from high salt purified THO complex. Western blot against Yra1 using an antibody directed against Yra1.
(TIF)

Figure S8 TREX is intact and recruited to genes at the 5′ end, but its occupancy does not increase towards the 3′ end of the gene in *THO2-TAP* cells. (A, B) TREX is recruited to the *PMA1* gene but its occupancy does not increase in the *THO2-TAP* mutant. To assess the occupancy of Hpr1 and Yra1 in the presence of the TAP-tag on Tho2, they were tagged with the Avi-tag. Occupancy of Hpr1 (A) and Yra1 (B) at the *PMA1* gene in a wt and the *THO2-TAP* strain. Results of 3 independent experiments are shown (mean +/− SD; **: p<0.01; *: p<0.05). (C) TAP-Tho2 and Tho2-TAP assemble into the TREX complex. TAP-Tho2 and Tho2-TAP were purified by tandem affinity purification. A Coomassie stain and Western blots against Yra1 and Sub2 of the whole cell extract (input, INP) and the calmodulin eluate (Cal-E) are shown.
(TIF)

Figure S9 The expression of gene classes other than length does not change in the THO2-TAP strain compared to a wt strain. Up- and down-regulated genes were analysed for their (A) expression level, (B) RNAPII levels, (C) GC-content, (D) convergent and (E) divergent gene spacing and for the positioning of the (F) +1 and (G) +2 nucleosome. No statistically significant effects were present in the *THO2-TAP* strain. The lines indicate the average of all genes, the bars represent the average of up- or down-regulated genes, respectively, the error bars indicate the SEM and the p-value was calculated using the Wilcox rank sum test. (H) Changes in expression of all genes were compared to SAGA or TFIID promoter dominated genes [61]. Bars represent the average of each gene class, the error bars indicate the SEM and the p-value was calculated using the Wilcox rank sum test.
(TIF)

Figure S10 *THO2-TAP* is synthetically lethal with *yra1-ΔPCID*. Growth of strains expressing Yra1 or yra1-ΔPCID and either no tagged protein, Hpr1-TAP or Tho2-TAP and carrying the plasmid pRS316-*YRA1* on SDC(-leu) and 5-FOA, which counterselects against the *URA3*-encoding pRS316 plasmid. *yra1-ΔPCID* is synthetically lethal with *THO2-TAP*, which causes an aberrant TREX occupancy profile, but not with *HPR1-TAP*.
(TIF)

Figure S11 The occupancy of transcription elongation factors binding to phosphorylated serine 2 or serine 5 does not increase during transcription elongation. (A) Meta gene occupancy profiles and (B) peak occupancy according to length classes for Spt6, Rtt103, Rna15, Pcf11 and Npl3 (S2P binders), Bur1 (S5P binder) and the TREX component Hpr1.
(TIF)

Table S1 Yeast strains used in this study.
(DOCX)

Table S2 Plasmids used in this study.
(DOCX)

Table S3 Sequences of the CTD peptides used in the pulldown experiments.
(DOCX)

Text S1 Supporting Materials and Methods and References.
(DOCX)
