# Supplementary figures and images for: Recruitment of TREX to the Transcription Machinery by Its Direct Binding to the Phospho-CTD of RNA Polymerase II
Source: PLoS Genet. 2013 Nov 14;9(11):e1003914. doi: 10.1371/journal.pgen.1003914 (PMC3828145; doi:10.1371/journal.pgen.1003914)

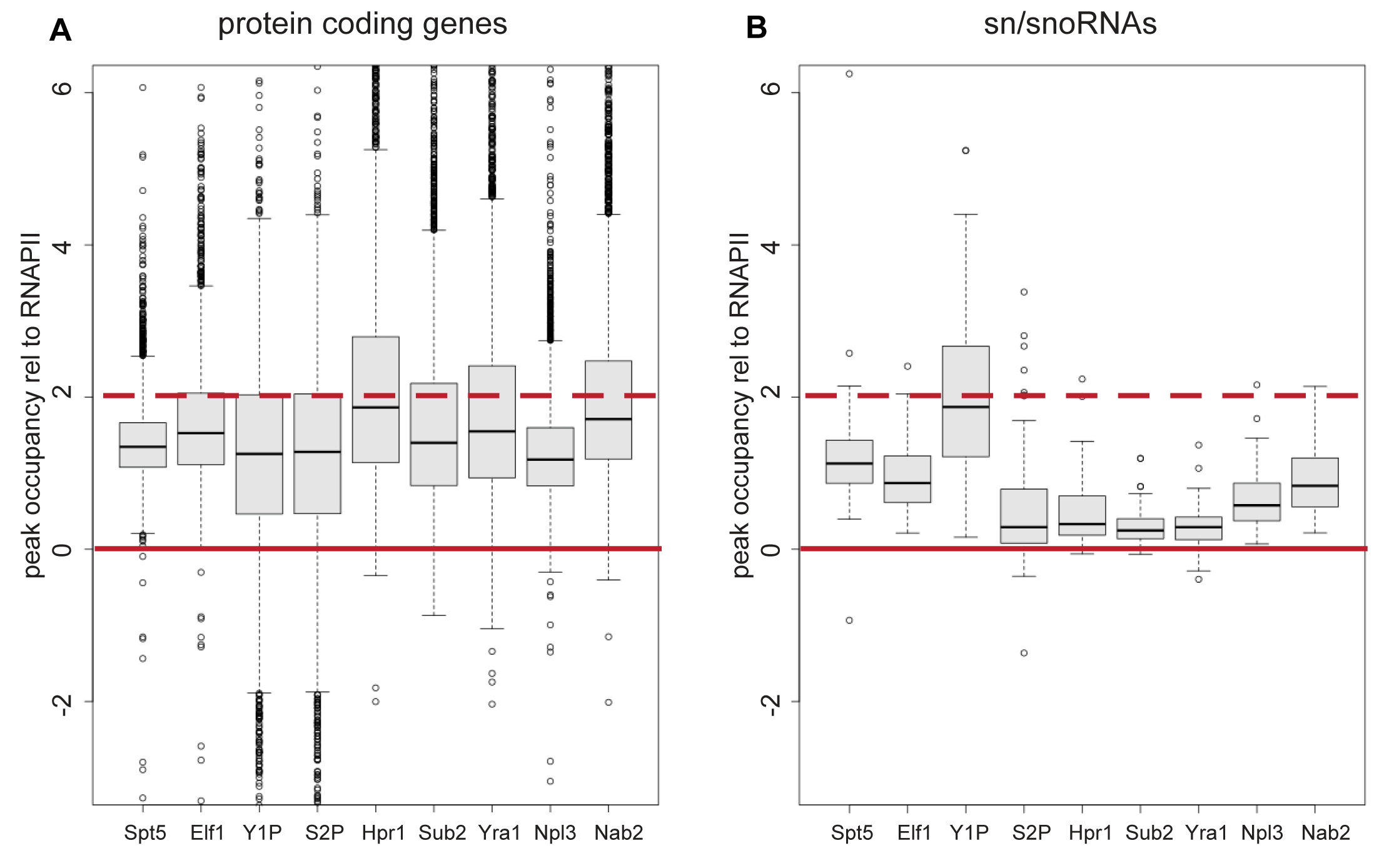

Supplement: Figure S1 — TREX is recruited to all RNAPII-transcribed genes. (A) Peak occupancies of the indicated proteins relative to RNAPII (Rpb3) on protein coding genes. The lower and upper borders of the boxes reflect the 25% and 75% quantiles, respectively, the black lines are the median values and the whiskers extend to the 1.5-fold inter quartile range. The red line gives the ratio 0, corresponding to no recruitment, and the dashed red line represents a ratio of 2. (B) Peak occupancies as in (A) but for sn/snoRNA genes. (TIF) [file pgen.1003914.s001.tif]

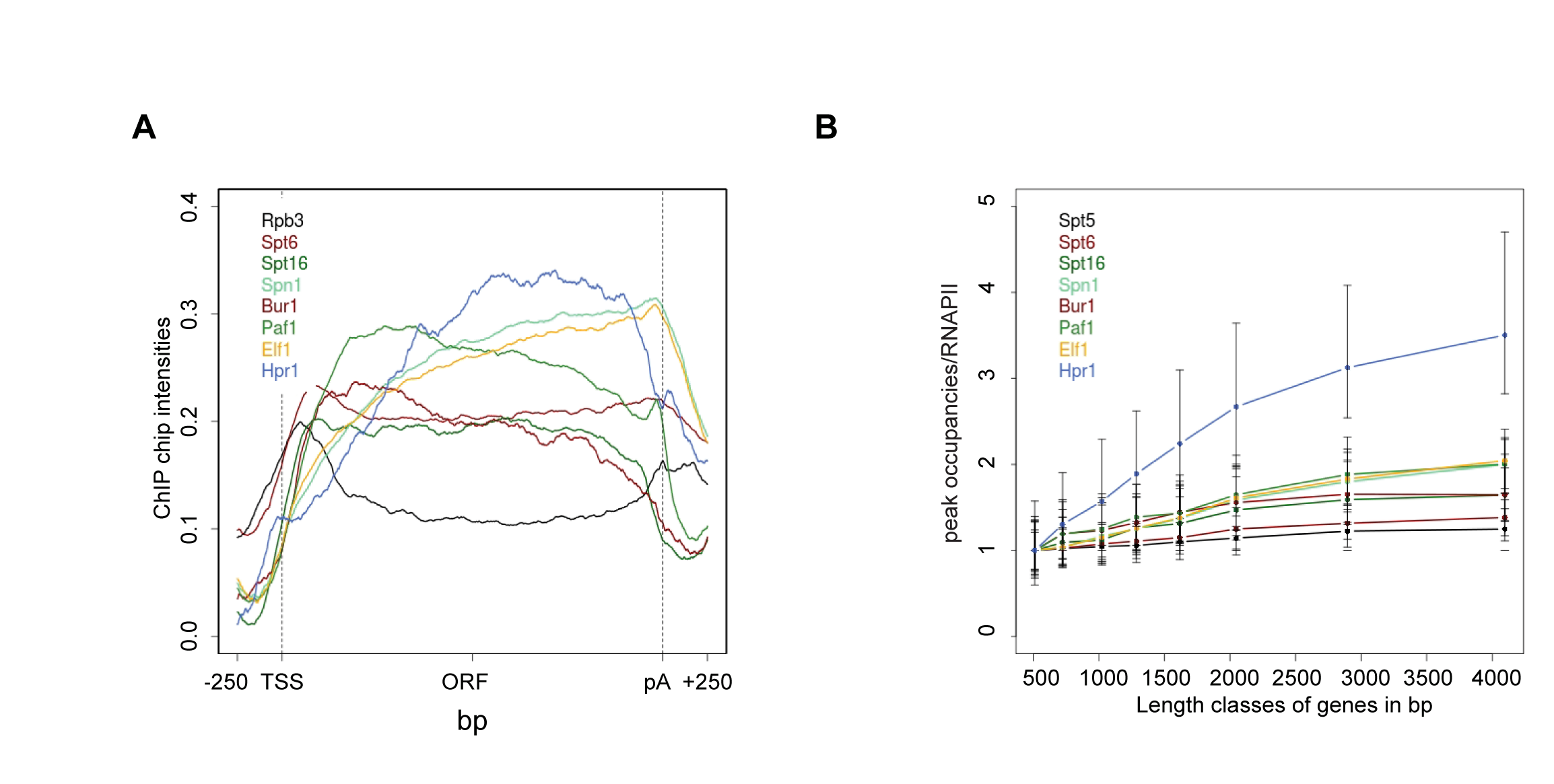

Supplement: Figure S2 — The occupancy of bona fide transcription elongation factors does not increase during transcription elongation. (A) Meta gene occupancy profiles and (B) peak occupancy according to length classes for RNAPII (Rpb3) (only A), the transcription elongation factors Spt5 (only B), Spt6, Spt16, Bur1 and Paf1 and the TREX component Hpr1. The peak occupancies in (B) were normalized to the peak occupancy of RNAPII to correct for the rate of transcription. (TIF) [file pgen.1003914.s002.tif]

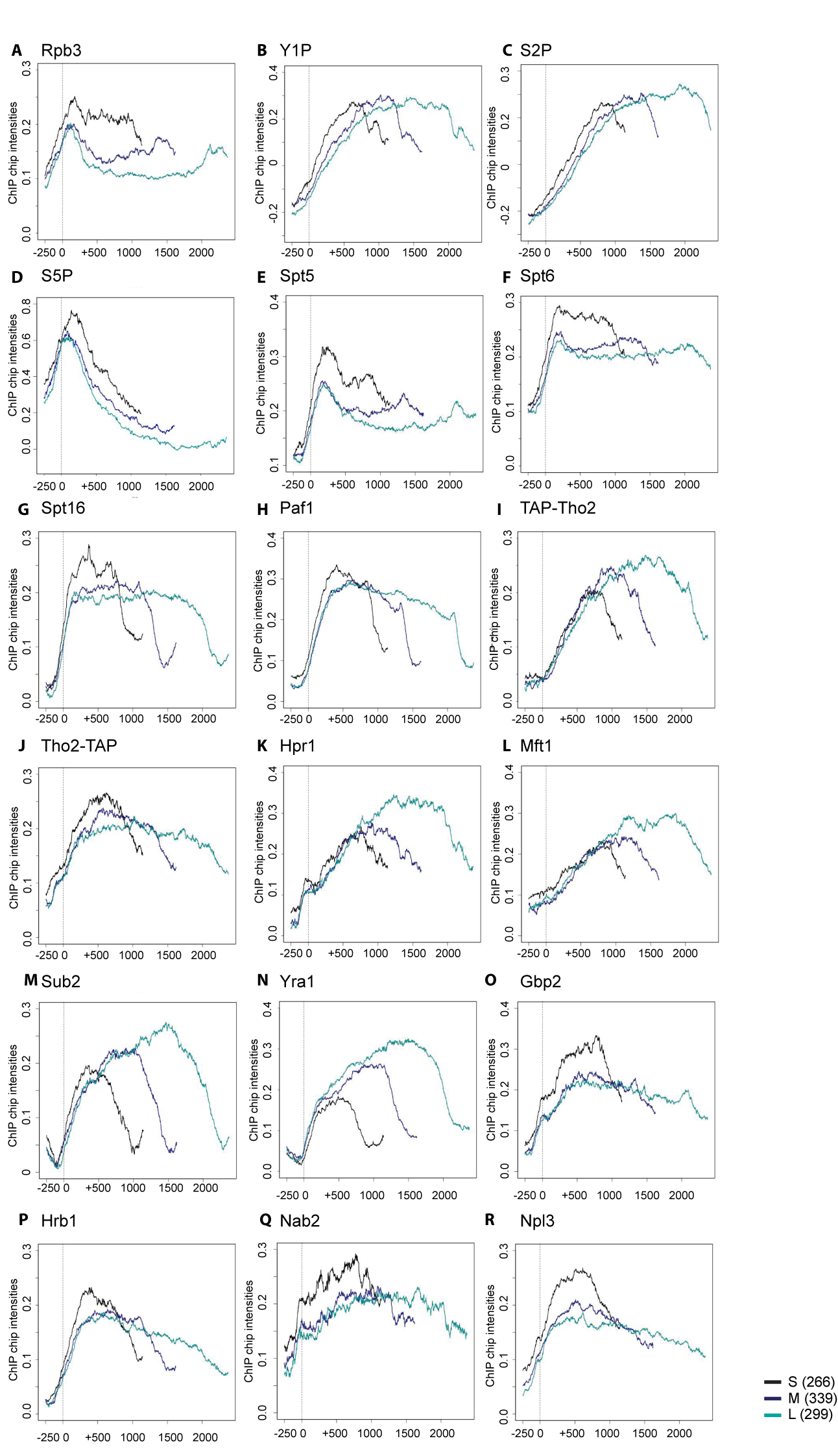

Supplement: Figure S3 — TREX occupancy increases with gene length. (A–Q) Meta gene occupancy profiles of RNAPII (Rpb3) (A), the CTD phosphomarks Y1P (B), S2P (C) and S5P (D), the transcription elongation factors Spt5 (E), Spt6 (F), Spt16 (G) and Paf1 (H), the THO components Tho2 (I), the mis-recruited allele Tho2-TAP (J), Hpr1 (K) and Mft1 (L), the TREX components Sub2 (M), Yra1 (N), Gbp2 (O) and Hrb1 (P) and the mRNA-binding proteins Nab2 (Q) and Npl3 (R) were calculated for different gene classes. The gene classes were defined as in [7]: S (512–937 bp, 266 genes), M (938–1537 bp, 339 genes) and L (1538–2895 bp, 299 genes). The number of genes in each class is given in parentheses. (TIF) [file pgen.1003914.s003.tif]

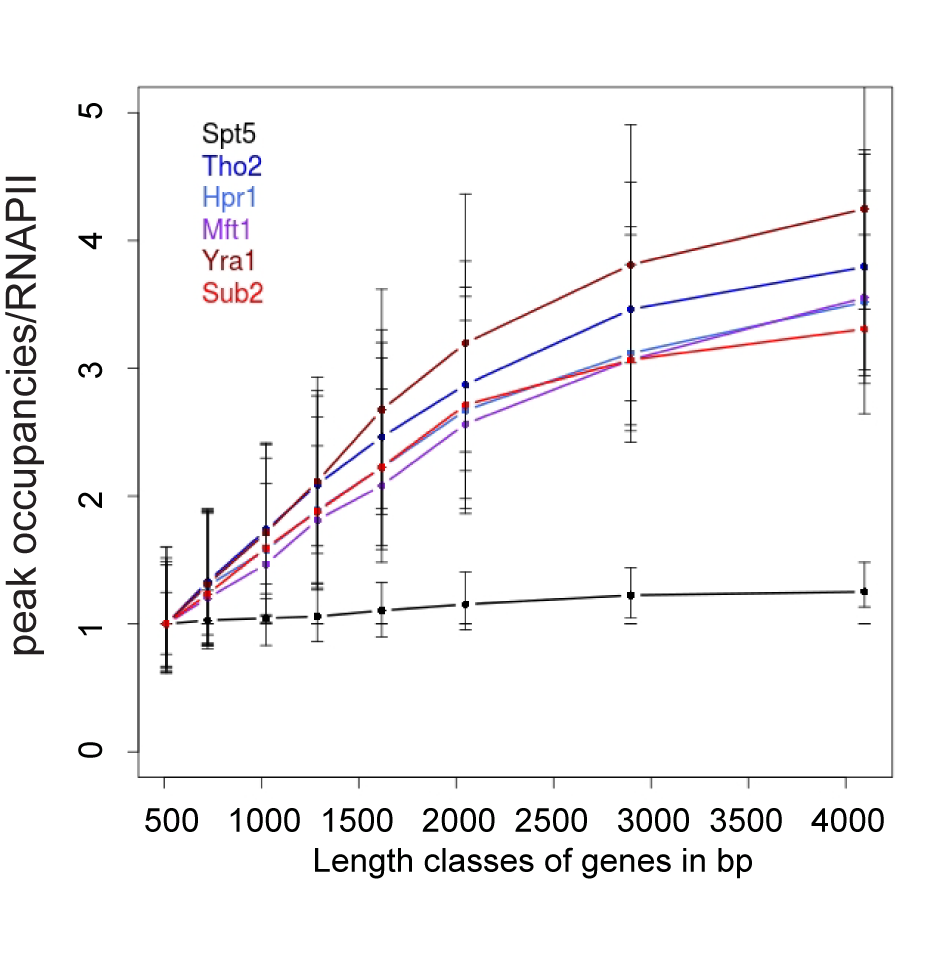

Supplement: Figure S4 — The 5′ to 3′ increase of TREX is independent of antisense transcription. The peak occupancy of TREX components Tho2, Hpr1, Mft1, Sub2 and Yra1 increases with gene length when genes containing a SUT or CUT 250 bp up- or downstream of the ORF were excluded from the calculation [60]. Calculations as for Figures 1B and S2. (TIF) [file pgen.1003914.s004.tif]

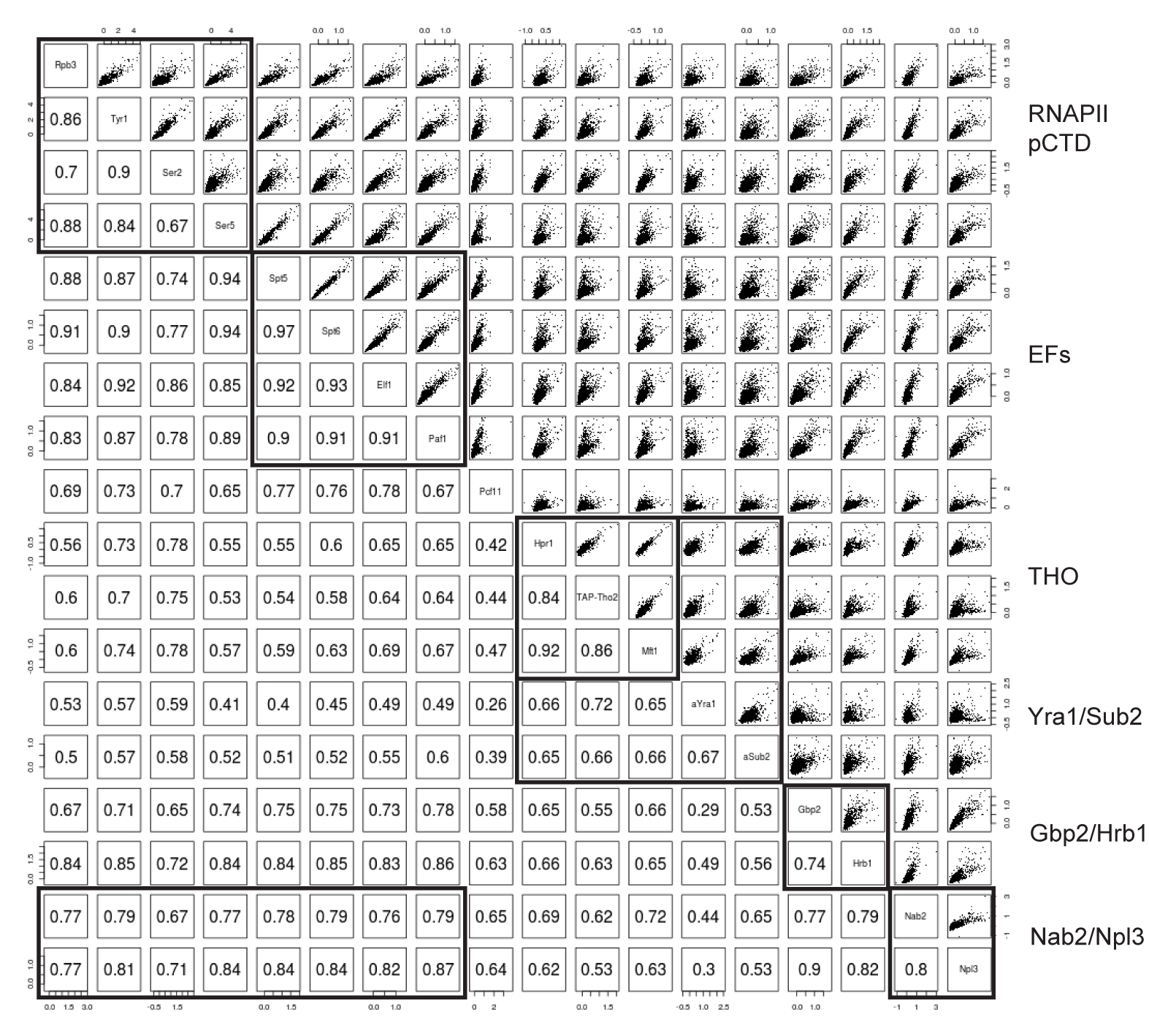

Supplement: Figure S5 — Pearson correlation coefficients between TREX components, Nab2, Npl3 and general elongation factors. The peak occupancies (90th percentile of each profile for each gene) of each protein were correlated for all protein coding genes. As expected, the general elongation factors Spt5, Spt6 and Elf1 correlate very highly with each other. Also RNAPII (Rpb3) and the phospho-CTD marks Y1P, S2P and S5P correlate very well with each other and with the general elongation factors. S2P strongly correlates with THO subunits, Yra1 and Sub2, and more weakly with general elongation factors. As expected, the THO subunits, Sub2 and Yra1 correlate highly with each other. Due to their lack of length dependency Gbp2 and Hrb1 correlate less with THO subunits, Sub2 and Yra1, but highly with Nab2 and Npl3. (TIF) [file pgen.1003914.s005.tif]

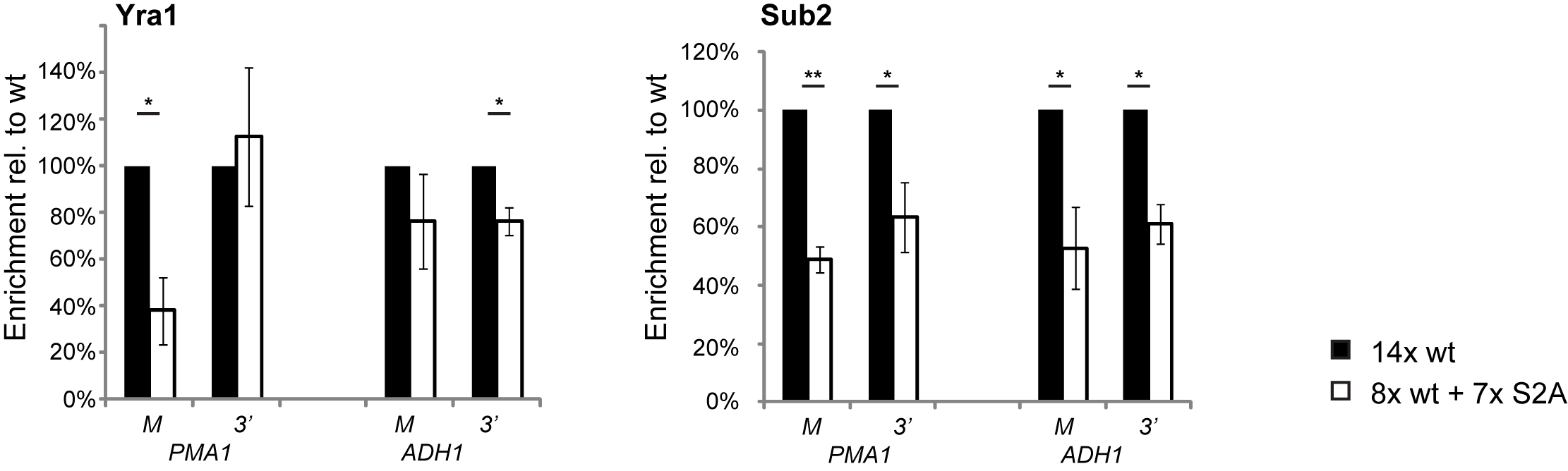

Supplement: Figure S6 — S2 phosphorylation is essential for recruitment of Sub2 and Yra1. The occupancies of Sub2 and Yra1 in the S2A mutant strain (white bars) were calculated relative to the occupancy in a strain with 14 wild-type CTD repeats (black bars). Results of at least 3 independent experiments are shown (mean +/− SD; **: p<0.01; *: p<0.05). (TIF) [file pgen.1003914.s006.tif]

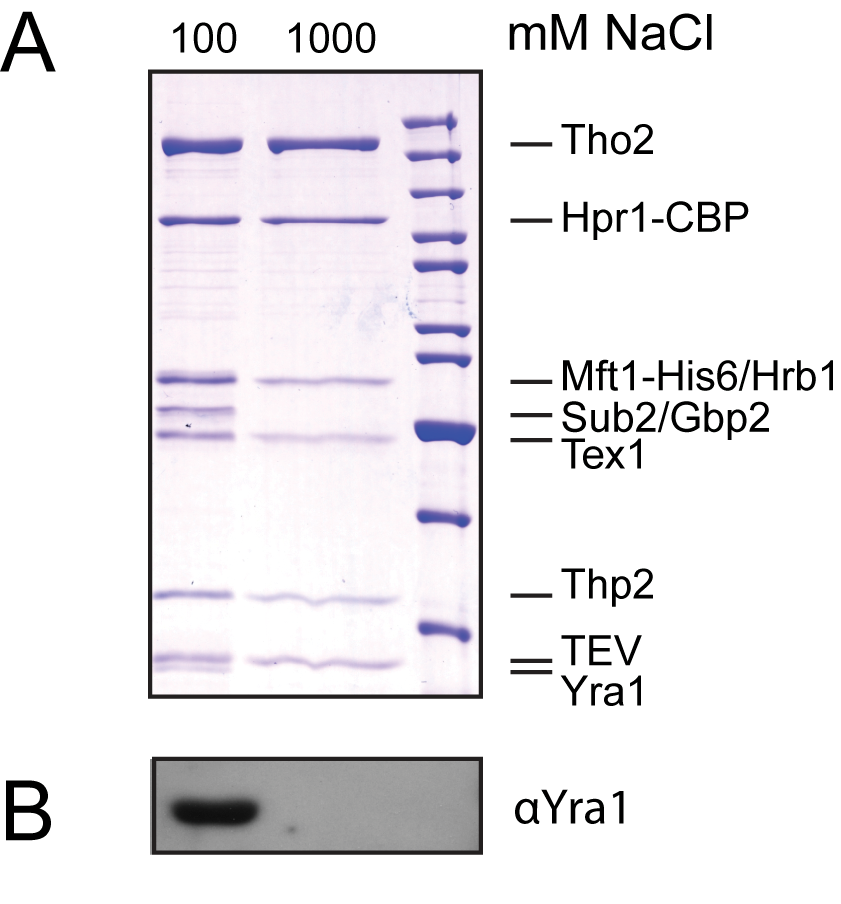

Supplement: Figure S7 — Tandem affinity purification (TAP) of the THO complex used in the CTD pulldown experiments. A strain expressing C-terminally TAP-tagged Hpr1 and C-terminally His6-tagged Mft1 was purified by two steps using IgG and Ni affinity purification under low salt (100 mM NaCl) and high salt (1000 mM NaCl) conditions yielding the whole TREX complex or the THO complex consisting of Tho2, Hpr1, Mft1, Thp2 and Tex1, respectively. (A) Coomassie stain of eluates after Ni affinity purification. The identity of each protein was verified by mass spectrometry and is indicated to the right. (B) Yra1 is absent from high salt purified THO complex. Western blot against Yra1 using an antibody directed against Yra1. (TIF) [file pgen.1003914.s007.tif]

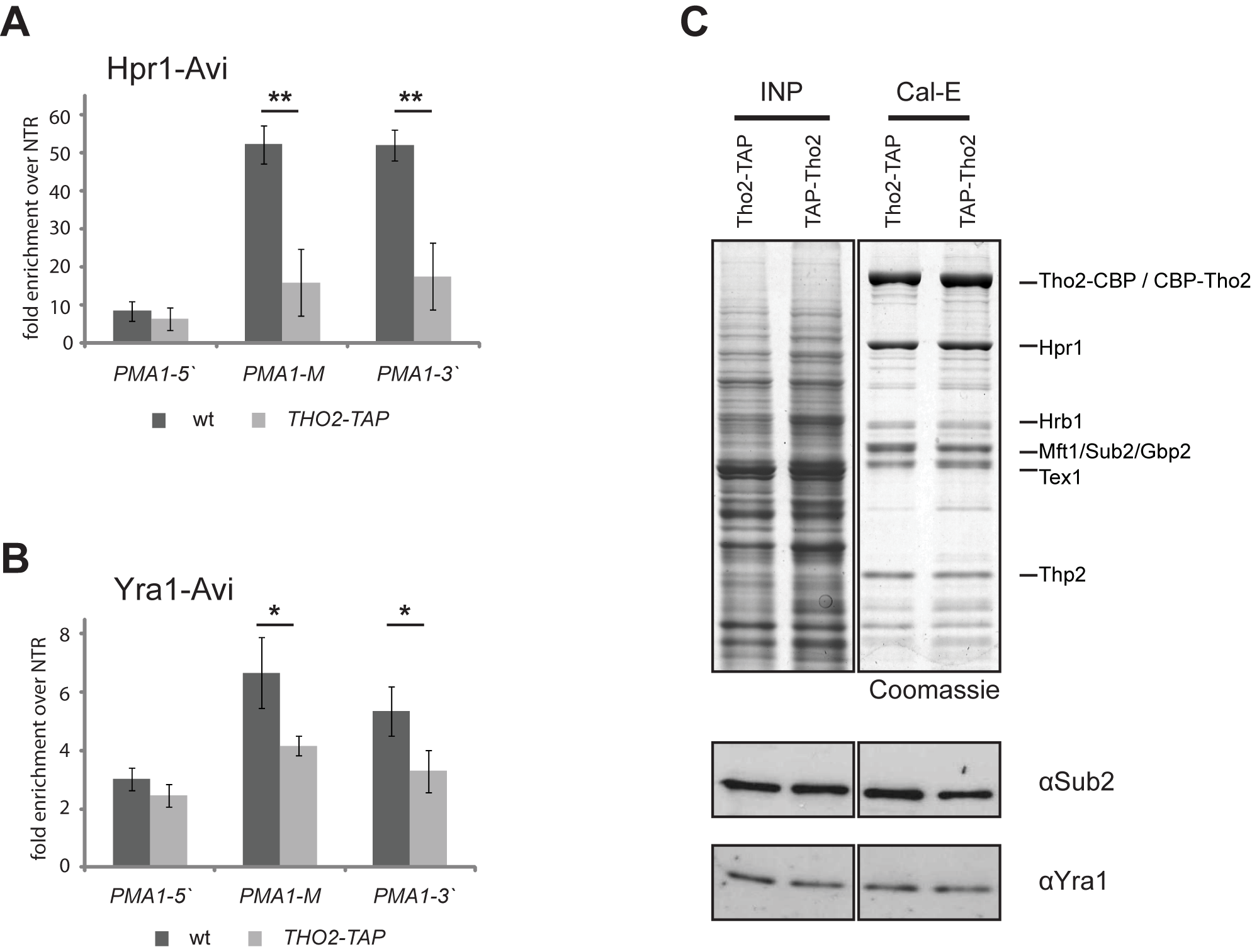

Supplement: Figure S8 — TREX is intact and recruited to genes at the 5′ end, but its occupancy does not increase towards the 3′ end of the gene in THO2-TAP cells. (A, B) TREX is recruited to the PMA1 gene but its occupancy does not increase in the THO2-TAP mutant. To assess the occupancy of Hpr1 and Yra1 in the presence of the TAP-tag on Tho2, they were tagged with the Avi-tag. Occupancy of Hpr1 (A) and Yra1 (B) at the PMA1 gene in a wt and the THO2-TAP strain. Results of 3 independent experiments are shown (mean +/− SD; **: p<0.01; *: p<0.05). (C) TAP-Tho2 and Tho2-TAP assemble into the TREX complex. TAP-Tho2 and Tho2-TAP were purified by tandem affinity purification. A Coomassie stain and Western blots against Yra1 and Sub2 of the whole cell extract (input, INP) and the calmodulin eluate (Cal-E) are shown. (TIF) [file pgen.1003914.s008.tif]

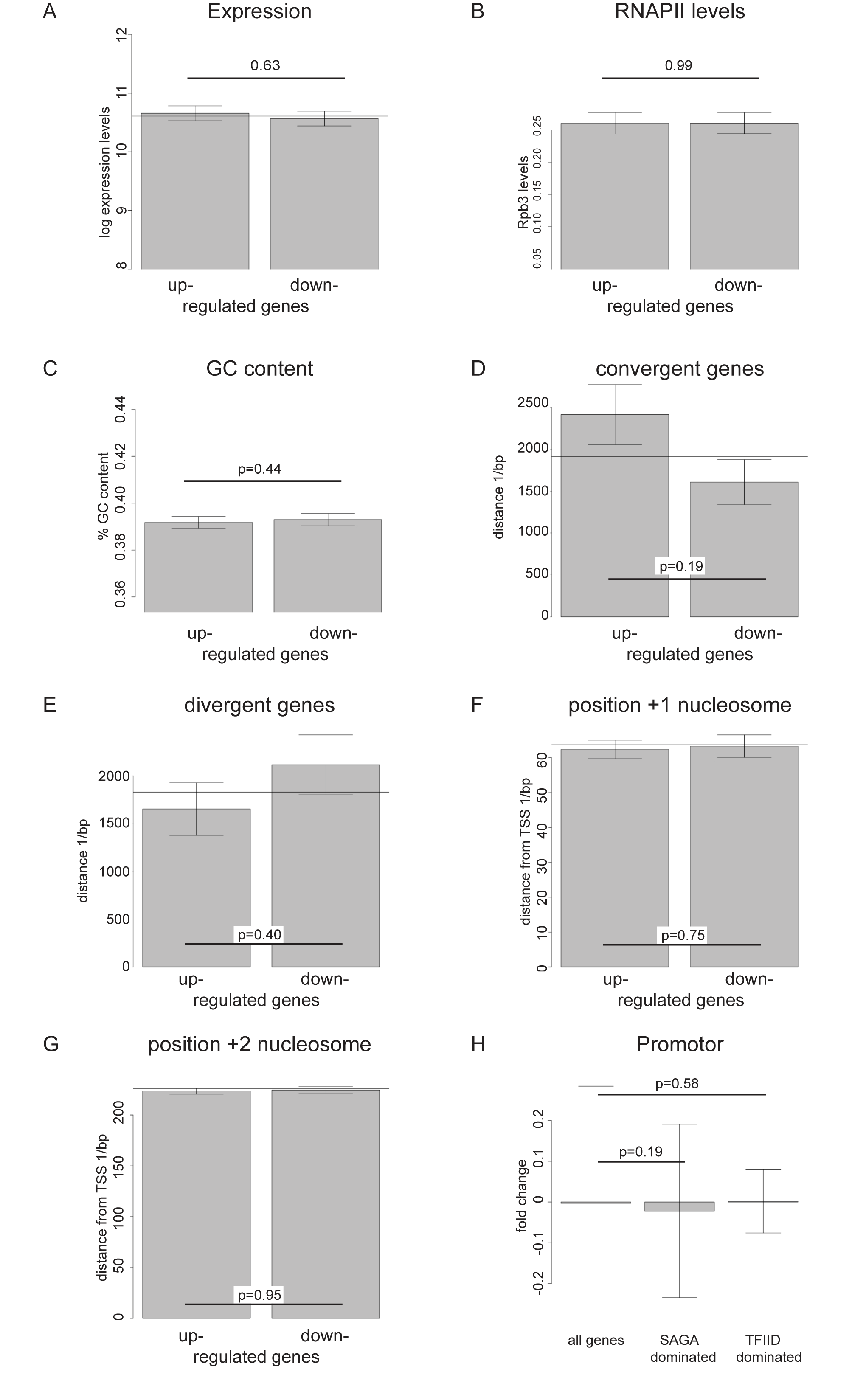

Supplement: Figure S9 — The expression of gene classes other than length does not change in the THO2-TAP strain compared to a wt strain. Up- and down-regulated genes were analysed for their (A) expression level, (B) RNAPII levels, (C) GC-content, (D) convergent and (E) divergent gene spacing and for the positioning of the (F) +1 and (G) +2 nucleosome. No statistically significant effects were present in the THO2-TAP strain. The lines indicate the average of all genes, the bars represent the average of up- or down-regulated genes, respectively, the error bars indicate the SEM and the p-value was calculated using the Wilcox rank sum test. (H) Changes in expression of all genes were compared to SAGA or TFIID promoter dominated genes [61]. Bars represent the average of each gene class, the error bars indicate the SEM and the p-value was calculated using the Wilcox rank sum test. (TIF) [file pgen.1003914.s009.tif]

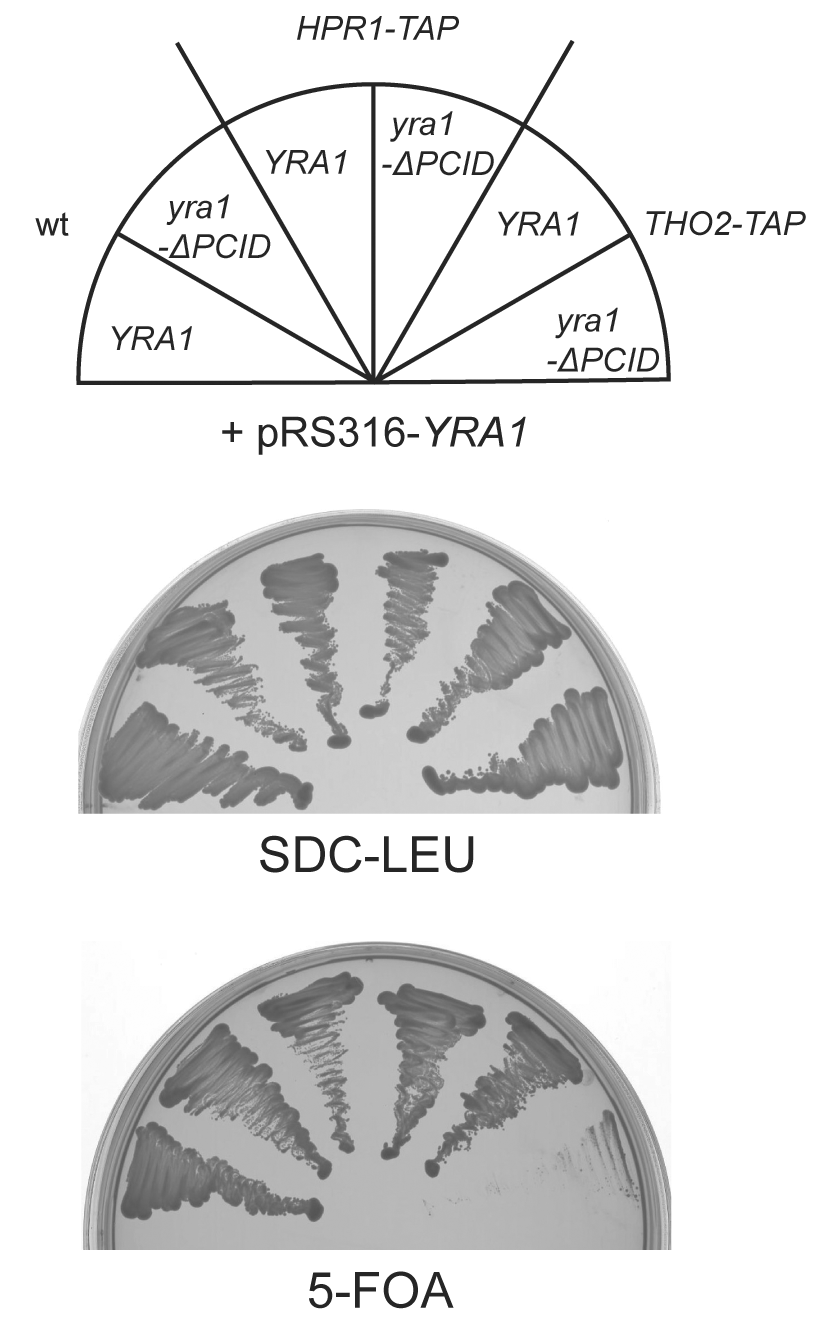

Supplement: Figure S10 — THO2-TAP is synthetically lethal with yra1-ΔPCID. Growth of strains expressing Yra1 or yra1-ΔPCID and either no tagged protein, Hpr1-TAP or Tho2-TAP and carrying the plasmid pRS316-YRA1 on SDC(-leu) and 5-FOA, which counterselects against the URA3-encoding pRS316 plasmid. yra1-ΔPCID is synthetically lethal with THO2-TAP, which causes an aberrant TREX occupancy profile, but not with HPR1-TAP. (TIF) [file pgen.1003914.s010.tif]

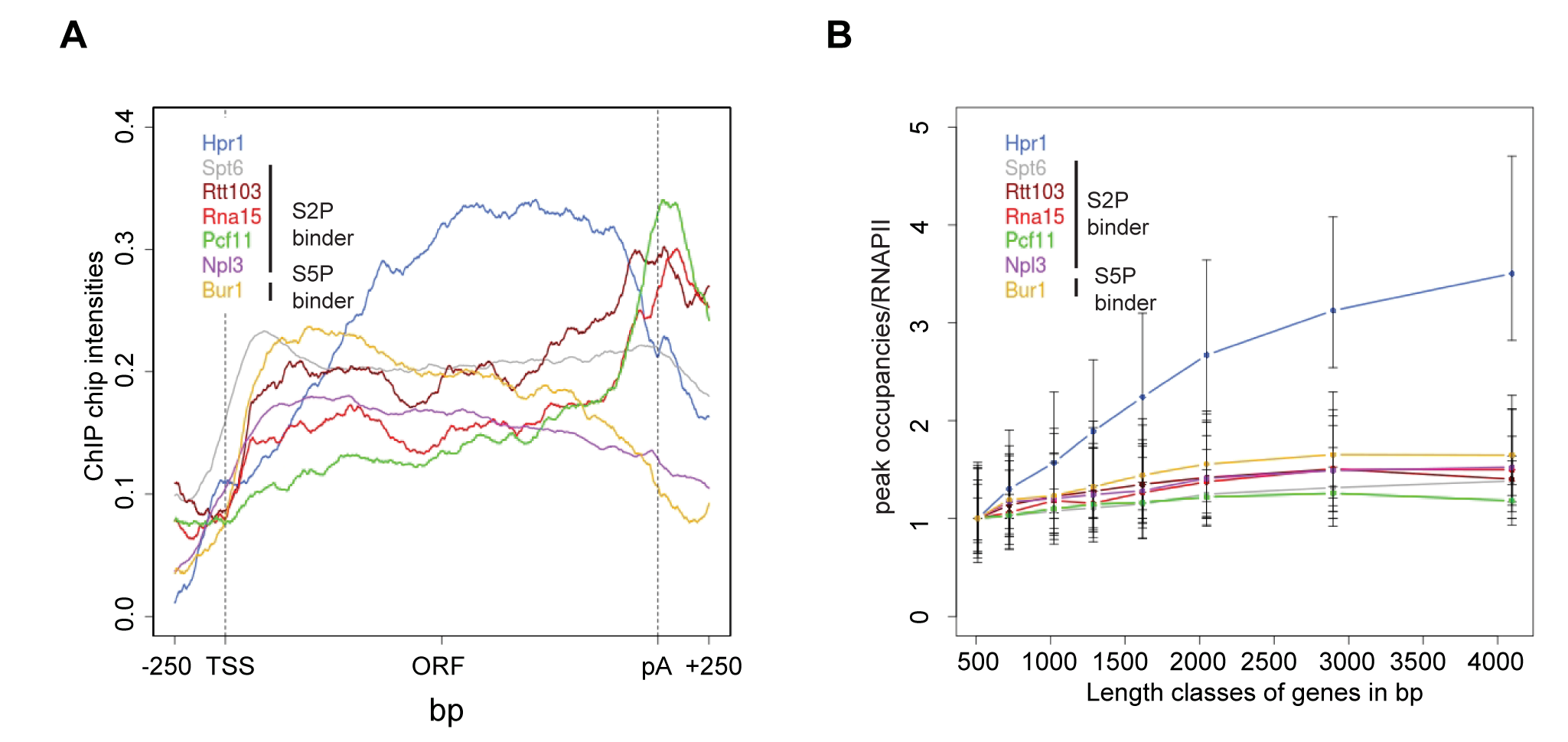

Supplement: Figure S11 — The occupancy of transcription elongation factors binding to phosphorylated serine 2 or serine 5 does not increase during transcription elongation. (A) Meta gene occupancy profiles and (B) peak occupancy according to length classes for Spt6, Rtt103, Rna15, Pcf11 and Npl3 (S2P binders), Bur1 (S5P binder) and the TREX component Hpr1. (TIF) [file pgen.1003914.s011.tif]
