# Supplementary material for: Recruitment of TREX to the Transcription Machinery by Its Direct Binding to the Phospho-CTD of RNA Polymerase II
Source: PLoS Genet. 2013 Nov 14;9(11):e1003914. doi: 10.1371/journal.pgen.1003914 (PMC3828145; doi:10.1371/journal.pgen.1003914)
Supplement: Table S1 — Yeast strains used in this study. (DOCX) [file pgen.1003914.s012.docx]

**Supporting Table 1.** Yeast strains used in this study.

| **Yeast strain** | **Genotype** | **Reference** |
| --- | --- | --- |
| BY4741 | MATa; *his3Δ1*; *leu2Δ0*; *met15Δ0*; *ura3Δ0* | Euroscarf |
| RS453 | MAT a; *ade2-1; his3-11,15; ura3-52; leu2-3,112; trp1-1; can1-100; GAL+* | ^1^ |
| *RPB3-TAP* | *RPB3-TAP::HIS3*; MATa; *his3Δ1*; *leu2Δ0*; *met15Δ0*; *ura3Δ0* | this study |
| *HPR1-TAP* | *HPR1-TAP::URA3*; MATa; *his3Δ1*; *leu2Δ0*; *met15Δ0*; *ura3Δ0* | this study |
| *TAP-THO2* | *TAP-THO2*, MATa; *his3Δ1*; *leu2Δ0*; *met15Δ0*; *ura3Δ0* | this study |
| *THO2-TAP* | *THO2-TAP::URA3*, MATa; *ade2-1; his3-11,15; ura3-52; leu2-3,112; trp1-1; can1-100; GAL+* | this study |
| *MFT1-TAP* | *MFT1-TAP::URA3*; MATa; *his3Δ1*; *leu2Δ0*; *met15Δ0*; *ura3Δ0* | this study |
| *GBP2-TAP* | *GBP2-TAP::URA3*; MATa; *his3Δ1*; *leu2Δ0*; *met15Δ0*; *ura3Δ0* | this study |
| *HRB1-TAP* | *HRB1-TAP::URA3*; MATa; *his3Δ1; leu2Δ0; met15Δ0; ura3Δ0* | this study |
| *TAP-NPL3* | *TAP::NPL3;* MATa; *his3Δ1; leu2Δ0; met15Δ0; ura3Δ0* | this study |
| *NAB2-TAP* | *NAB2-TAP::URA3;* MATa; *his3Δ1; leu2Δ0; met15Δ0; ura3Δ0* | this study |
| *HPR1-TAP MFT1-His_6_* | *HPR1-TAP::TRP1; MFT1-myc-His6::HIS3;* MAT alpha; *ade2-1; his3-11,15; ura3-52; leu2-3,112; trp1-1; can1-100; GAL+* | this study |
| *RIX1-TAP* | *RIX1-TAP::TRP1;* MAT alpha; *ade2-1; his3-11,15; ura3-52; leu2-3,112; trp1-1; can1-100; GAL+* | this study |
| *PCF11-TAP* | *PCF11-TAP::TRP1;* MAT a; *ade2-1; his3-11,15; ura3-52; leu2-3,112; trp1-1; can1-100; GAL+* | this study |
| wt, σ active | *Ribo-YCT1::KanMX* ; MATa*; his3Δ1; leu2Δ0; met15Δ0; ura3Δ0* | this study |
| wt, σ inactive | *RiboMut-YCT1::KanMX* ;MATa; *his3Δ1; leu2Δ0; met15Δ 0; ura3Δ0* | this study |
| *RPB3-TAP*  σ active | *RPB3-TAP::HIS3;* *Ribo-YCT1::KanMX*; MATa*; his3Δ1; leu2Δ0; met15Δ0; ura3Δ0* | this study |
| *RPB3-TAP* σ inactive | *RPB3-TAP::HIS3; RiboMut-YCT1::KanMX* ;MATa; *his3Δ 1; leu2Δ0; met15Δ0; ura3Δ0* | this study |
| *HPR1-TAP*  σ active | *HPR1-TAP::URA3,Ribo-YCT1::KanMX*; MATa; *his3Δ1; leu2Δ0; met15Δ0; ura3Δ0* | this study |
| *HPR1-TAP*  σ inactive | *HPR1-TAP::URA3, RiboMut-YCT1::KanMX*; MATa; *his3Δ 1; leu2Δ0; met15Δ0; ura3Δ0* | this study |
| *TAP-THO2*  σ active | *TAP-THO2, Ribo-YCT1::KanMX ;*MATa; *his3Δ1; leu2Δ0; met15Δ0; ura3Δ0* | this study |
| *TAP-THO2*  σ inactive | *TAP-THO2;RiboMut-YCT1::KanMX*; MATa; *his3Δ1; leu2Δ 0; met15Δ0; ura3Δ0* | this study |
| *THP2-TAP*  σ active | *THP2-TAP::URA3; Ribo-YCT1::KanMX*; MATa; *his3Δ1; leu2Δ0; met15Δ0; ura3Δ0* | this study |
| *THP2-TAP*  σ inactive | *THP2-TAP::URA3; RiboMut-YCT1::KanMX*; MATa; *his3Δ1; leu2Δ0; met15Δ0; ura3Δ0* | this study |
| *MFT1-TAP*  σ active | *MFT1-TAP::URA3; Ribo-YCT1::KanMX*; MATa; *his3Δ1; leu2Δ0; met15Δ0; ura3Δ0* | this study |
| *MFT1-TAP*  σ inactive | *MFT1-TAP::URA3; RiboMut-YCT1::KanMX*; MATa; *his3Δ1; leu2Δ0; met15Δ0; ura3Δ0* | this study |
| *HRB1-TAP*  σ active | *HRB1-TAP::URA3; Ribo-YCT1::KanMX*; MATa; *his3Δ1; leu2Δ0; met15Δ0; ura3Δ0* | this study |
| *HRB1-TAP*  σ inactive | *HRB1-TAP::URA3; RiboMut-YCT1::KanMX*; MATa; *his3Δ1; leu2Δ0; met15Δ0; ura3Δ0* | this study |
| *GBP2-TAP*  σ active | *GBP2-TAP::URA3; Ribo-YCT1::KanMX;* MATa; *his3Δ1; leu2Δ0; met15Δ0; ura3Δ0* | this study |
| *GBP2-TAP*  σ inactive | *GBP2-TAP::URA3; RiboMut-YCT1::KanMX*; MATa; *his3Δ1; leu2Δ0; met15Δ0; ura3Δ0* | this study |
| *NAB2-TAP*  σ active | *NAB2-TAP::URA3; Ribo-YCT1::KanMX*; MATa; *his3Δ1; leu2Δ0; met15Δ0; ura3Δ0* | this study |
| *NAB2-TAP*  σ inactive | *NAB2-TAP::URA3; RiboMut-YCT1::KanMX*; MATa; *his3Δ1; leu2Δ0; met15Δ0; ura3Δ0* | this study |
| *TAP-NPL3*  σ active | *TAP::NPL3; Ribo-YCT1::KanMX;* MATa; *his3Δ1; leu2Δ0; met15Δ0; ura3Δ0* | this study |
| *TAP-NPL3*  σ inactive | *TAP::NPL3; RiboMut-YCT1::KanMX*; MATa; *his3Δ1; leu2Δ0; met15Δ0; ura3Δ0* | this study |
| *YRA1* shuffle | *yra1::HIS3*; MAT a; *ade2-1*; *his3-11,15*; *ura3-52*; *leu2-3,112*; trp1-1; can1-100; GAL*+;* pRS316*-YRA1* | 1 |
| *YRA1* shuffle *HPR1-TAP* | *yra1::HIS3; HPR1-TAP::TRP1;* MAT a; *ade2-1; his3-11,15; ura3-52; leu2-3,112; trp1-1; can1-100; GAL+;* pRS316*-YRA1* | this study |
| *YRA1* shuffle *THO2-TAP* | *yra1::HIS3; THO2-TAP::TRP1;* MAT a; *ade2-1; his3-11,15; ura3-52; leu2-3,112; trp1-1; can1-100; GAL+;* pRS316*-YRA1* | this study |
| *RPB1 shuffle* | *rpb1::HIS3; GAL1::YLR454::TRP1;* MAT a; *ade2-1; his3-11,15; ura3-52; leu2-3,112; trp1-1; can1-100; GAL+,* pRS316*-RPB1* | this study |
| *RPB1 shuffle* *HPR1-TAP* | *rpb1::HIS3; HPR1-TAP::KanMX, GAL1::YLR454::TRP1*, MAT a; *ade2-1; his3-11,15; ura3-52; leu2-3,112; trp1-1; can1-100; GAL+,* pRS316*-RPB1* | this study |
| *Hpr1-Avi,*  pRS315*-BirA-NLS* | *HPR1-Avi::KanMX, MAT a; ade2-1; his3-11,15; ura3-52; leu2-3,112; trp1-1; can1-100; GAL+,* pRS315*-BirA-NLS* | this study |
| *Hpr1-Avi,*  *Tho2-TAP*  pRS315*-BirA-NLS* | *HPR1-Avi::KanMX, THO2-TAP::TRP1KL, MAT a; ade2-1; his3-11,15; ura3-52; leu2-3,112; trp1-1; can1-100; GAL+,* pRS315*-BirA-NLS* | this study |
| *SUB2-Avi,*  pRS315*-BirA-NLS* | *SUB2-Avi::KanMX, MAT a; ade2-1; his3-11,15; ura3-52; leu2-3,112; trp1-1; can1-100; GAL+,* pRS315*-BirA-NLS* | this study |
| *SUB2-Avi,*  *Tho2-TAP*  pRS315*-BirA-NLS* | *SUB2-Avi::KanMX, THO2-TAP::TRP1KL, MAT a; ade2-1; his3-11,15; ura3-52; leu2-3,112; trp1-1; can1-100; GAL+,* pRS315*-BirA-NLS* | this study |
| *YRA1-Avi,*  pRS315*-BirA-NLS* | *YRA1-Avi::KanMX, MAT a; ade2-1; his3-11,15; ura3-52; leu2-3,112; trp1-1; can1-100; GAL+,* pRS315*-BirA-NLS* | this study |
| *YRA1-Avi,*  *Tho2-TAP*  pRS315*-BirA-NLS* | *YRA1-Avi::KanMX, THO2-TAP::TRP1KL, MAT a; ade2-1; his3-11,15; ura3-52; leu2-3,112; trp1-1; can1-100; GAL+,* pRS315*-BirA-NLS* | this study |
| *RPB1-Avi,*  pRS315*-BirA-NLS* | *RPB1-Avi::KanMX, MAT a; ade2-1; his3-11,15; ura3-52; leu2-3,112; trp1-1; can1-100; GAL+,* pRS315*-BirA-NLS* | this study |
| *RPB1-Avi,*  *THO2-TAP*  pRS315*-BirA-NLS* | *RPB1-Avi::KanMX, THO2-TAP::TRP1KL, MAT a; ade2-1; his3-11,15; ura3-52; leu2-3,112; trp1-1; can1-100; GAL+,* pRS315*-BirA-NLS* | this study |

^1^ Strasser K & Hurt E (2000) Yra1p, a conserved nuclear RNA-binding protein, interacts directly with Mex67p and is required for mRNA export. Embo J 19, 410-20
