# Supplementary material for: Recruitment of TREX to the Transcription Machinery by Its Direct Binding to the Phospho-CTD of RNA Polymerase II
Source: PLoS Genet. 2013 Nov 14;9(11):e1003914. doi: 10.1371/journal.pgen.1003914 (PMC3828145; doi:10.1371/journal.pgen.1003914)
Supplement: Table S2 — Plasmids used in this study. (DOCX) [file pgen.1003914.s013.docx]

**Supporting Table 2.** Plasmids used in this study.

| **Plasmid** | **Description** | **Reference** |
| --- | --- | --- |
| pY1WT | *rpb1-14xwt repeats* | ^1^ |
| pY1WT(7)A9(6) | *rpb1*-9xwt+5xS2A | ^1^ |
| pY1WT(5)F1(9) | *rpb1*-5xwt+9xY1F | this study |
| pRS315-3HA-*YRA1* | 3HA-Yra1(1-226) | this study |
| pRS315-3HA-*yra1-ΔPCID* | 3HA-Yra1-ΔPCID(aa 77-226) | this study |
| pRibo-Active | *GAL1::GFP*-active σ ribozyme | this study |
| pRibo-Inactive | *GAL1::GFP* -inactive σ ribozyme | this study |
| pRS315*-BirA-NLS* | BirA biotin ligase fused to a nuclear localization sequence | ^2^ |

^1^ West ML & Corden JL (1995) Construction and analysis of yeast RNA polymerase II CTD deletion and substitution mutations. Genetics 140, 1223-33

^2^ van Werven FJ & Timmers HT(2006) The use of biotin tagging in Saccharomyces cerevisiae improves the sensitivity of chromatin immunoprecipitation. Nucleic acids research 34, e33
