# Supplementary material for: Recruitment of TREX to the Transcription Machinery by Its Direct Binding to the Phospho-CTD of RNA Polymerase II
Source: PLoS Genet. 2013 Nov 14;9(11):e1003914. doi: 10.1371/journal.pgen.1003914 (PMC3828145; doi:10.1371/journal.pgen.1003914)
Supplement: Table S3 — Sequences of the CTD peptides used in the pulldown experiments. (DOCX) [file pgen.1003914.s014.docx]

**Supporting Table 3.** Sequences of the CTD peptides used in the pulldown experiments.

| **Peptide** | **Sequence** | **Company** |
| --- | --- | --- |
| CTD | Biotin - YSPTSPS YSPTSPS YSPTSPS | PSL, Heidelberg |
| S2P | Biotin - YSPTSPS Y**pS**PTSPS Y**pS**PTSPS | PSL, Heidelberg |
| S5P | Biotin - YSPTSPS YSPT**pS**PS YSPT**pS**PS | PSL, Heidelberg |
| Y1P | Biotin - YSPTSPS **pY**SPTSPS **pY**PTSPS | PSL, Heidelberg |
| Y1P-S2P | Biotin - YSPTSPS **pYpS**PTSPS pYpSPTSPS | PANATECS, Tübingen |
| Y1P-S5P | Biotin - YSPTSPS **pY**SPT**pS**PS **pY**SPT**pS**PS | PANATECS, Tübingen |
| S2P-S5P | Biotin - YSPTSPS Y**pS**PT**pS**PS Y**pS**PT**pS**PS | PANATECS, Tübingen |

**pY**: phospho-tyrosine; **pS**: phospho-serine
