# Supplementary material for: Recruitment of TREX to the Transcription Machinery by Its Direct Binding to the Phospho-CTD of RNA Polymerase II
Source: PLoS Genet. 2013 Nov 14;9(11):e1003914. doi: 10.1371/journal.pgen.1003914 (PMC3828145; doi:10.1371/journal.pgen.1003914)
Supplement: Text S1 — Supporting Materials and Methods and References. (DOCX) [file pgen.1003914.s015.docx]

**Supporting Materials and Methods**

**Yeast strains**

The integration of an N- or C-terminal TAP tag was performed according to [[1](#_ENREF_1)]. The Avi-tag was integrated as in [[2](#_ENREF_2)]. The ribozyme reporter constructs were integrated into yeast by amplifying the *GAL1::GFP*-σ-ribozyme construct with primers containing approximately 50 bp homologous to the region upstream and downstream of the start codon of *YCT1*.

**Plasmids**

Plasmids encoding Yra1 and ΔPCID-Yra1 were cloned as the plasmids published in [[3](#_ENREF_3)], but with the endogenous *YRA1* promoter. The promoter of *YRA1* (-333 to 8 nt relative to the *YRA1* start codon) was amplified from genomic DNA with primers encoding an HA_3_-tag added and inserted SacI and XhoI into pRS313. *YRA1* (encoding aa 1-226) and *yra1-ΔPCID* (encoding aa 77-226) were amplified including the *YRA1* terminator sequence (621 bp) from genomic DNA and cloned XhoI and ApaI into the above construct. These two *YRA1* constructs were subcloned SacI and ApaI into pRS315. Plasmids encoding *RPB1* with the truncated and mutated versions of the CTD were cloned similar to those published by [[4](#_ENREF_4)], except that primers coding for 7 repeats were used. To clone the constructs for integration of the ribozyme sequence the selection marker of pYM-N25 [[5](#_ENREF_5)] was exchanged by the KanMX cassette of pYM-N22 by standard cloning techniques. Templates Ribo-active and Ribo-inactive for the ribozymes were synthesized, PCR amplified and cloned EcoRI and EcoRV into the modified pYM-N25.

**ChIP experiments**

ChIP was essentially performed as described in [[6](#_ENREF_6)]. Briefly, cells were lysed with equal volumes of glass beads for 5 times 3 min with 3 min breaks on ice. After sonication, which yielded an average chromatin size of 250 bp, the cleared lysate was incubated with 15 µl IgG coupled magnetic beads (Invitrogen). For ChIPs using antibodies, lysates were incubated for 2 h (20°C) with 50 µl αY1P, 20 µl αS2P[[7](#_ENREF_7)], αRpb1 (Y-80, Santa Cruz), αYra1[[8](#_ENREF_8)] or αSub2[[8](#_ENREF_8)] antibodies and 1.5 h (20°C) with 15 µl Protein A or G coupled magnetic beads (Invitrogen). After washing, elution and Proteinase K digestion, DNA was purified using a PCR clean up Kit (Machery-Nagel). The Avi-tag ChIPs were performed as described in [[2](#_ENREF_2)]. The DNA was analysed by quantitative PCR. An Applied Biosystems Step One Plus cycler was used with the Applied Biosystems Power Sybr Green PCR Master Mix. A non-transcribed region (NTR) on Chromosome V (174131-174200) served as negative control. Standard curves were used to estimate primer efficiencies and factor occupancies were calculated relative to the non-transcribed region according to (E^(C_T_^IP^-C_T_^INP^))_NTR_/(E ^(C_T_^IP^-C_T_^INP^)).

**ChIP-chip experiments and data analysis**

ChIP-chip experiments and data analysis were essentially performed as in [[7](#_ENREF_7),[9](#_ENREF_9)]. For ChIP-chip experiments 2-4 replicates were performed for each protein. Briefly, TAP-tagged proteins were normalized to the mock as well as input samples. The 99.8% quantile of the normalized signal was set to 100% and the 10% quantile was set to 0% to account for background. The occupancy for each nucleotide was calculated by the median of the overlapping probes at that position. Values for single outlier probes, not correlating well between the replicates, were removed and interpolated by the neighbouring, overlapping probes on the tilling array. Profiles were smoothed with a running window half size of 75 bp. Genes were filtered by annotated genes with known transcriptional start sites (TSS) and polyA sites (polyA). For the calculation of meta gene occupancy profiles the 50% most highly transcribed genes of all gene classes that were at least 200 nt separated from neighbouring genes were used for analysis. Different open reading frame sizes were defined. For the meta gene occupancy profiles in Figures 1, 3, 6 and S2 genes of 1,538-2,895 bp in length, comprising 299 genes, were used. In Figure S3 gene classes were defined as in [[7](#_ENREF_7)]: S (512-937bp, 266 genes), M (938-1537 bp, 339 genes) and L (1538—2895 bp, 299 genes). Profiles within each gene group were scaled to median gene length and the median signal was plotted for each position. For the length plots in Figures 1, 3, 6, S2, S4 and S11 all genes that were separated by at least 200 bp from neighbouring genes were used and the following eight length classes were defined: A (512-723bp), B (724-1023 bp), C (1024-1286 bp), D (1287-1617 bp), E (1618-2047 bp), F (2048-2895 bp), G (2896-4095 bp) and H (4096-5793 bp). Peak occupancies are the 90% quantile value for the profile of the according protein over each gene, which gives a robust measure for the maximum recruitment of each protein to the gene. The profiles used for the peak occupancies consist of the coding region and 100 nt up and downstream of each gene and were normalized to the peak occupancy of RNAPIII. The ChIP data to calculate the meta gene occupancy profiles and peak occupancies for Spt5, S2P, Y1P, S5P, Spt6, Rtt103, Rna15, Pcf11, Bur1, Spt16, Spn1, Paf1 and Elf1 were taken from [[7](#_ENREF_7)], [[9](#_ENREF_9)] and [[10](#_ENREF_10)].

**Tandem Affinity Purification**

Tandem affinity purifications (TAP) were performed essentially as described in [[8](#_ENREF_8)]. For the preparation of protein complexes used in the pull down assays 2 l of OD 3-3.6 yeast were lysed with glass beads. The cleared lysate was bound for 2 h at 4°C to 400 µl IgG Sepharose beads (GE). After washing with 250 mM NaCl TAP Buffer (50 mM Tris/HCl, pH 7.5, 1.5 mMgCl_2_, 0.15% NP-40, 1 mM DTT), for Rix1-TAP and Pcf11-TAP, 100 mM NaCl TAP-buffer for TREX (Hpr1-TAP + Mft1-His_6_) or 1000 mM NaCl TAP-buffer for THO (Hpr1-TAP + Mft1-His_6_) protein complexes were eluted using 150 µl 100 mM NaCl TAP-buffer and TEV protease by incubation for 90 min at 16°C. THO and TREX were bound to 250 µl Ni-NTA Agarose (Quiagen) in 1000 mM / 100 mM NaCl TAP buffer, respectively, including 20 mM imidazole for 1 h at 4°C, washed with 100 mM NaCl TAP-buffer (TREX) or 1000 mM NaCl TAP-buffer (THO) and eluted in 2x 75 µl elution buffer (100 mM NaCl TAP-buffer, 250 µM imidazole).

**Supporting References**

1. Rigaut G, Shevchenko A, Rutz B, Wilm M, Mann M, et al. (1999) A generic protein purification method for protein complex characterization and proteome exploration. Nat Biotechnol. pp. 1030-1032.

2. van Werven FJ, Timmers HT (2006) The use of biotin tagging in Saccharomyces cerevisiae improves the sensitivity of chromatin immunoprecipitation. Nucleic acids research 34: e33.

3. MacKellar AL, Greenleaf AL (2011) Cotranscriptional association of mRNA export factor Yra1 with C-terminal domain of RNA polymerase II. The Journal of biological chemistry 286: 36385-36395.

4. West ML, Corden JL (1995) Construction and analysis of yeast RNA polymerase II CTD deletion and substitution mutations. Genetics 140: 1223-1233.

5. Janke C, Magiera MM, Rathfelder N, Taxis C, Reber S, et al. (2004) A versatile toolbox for PCR-based tagging of yeast genes: new fluorescent proteins, more markers and promoter substitution cassettes. Yeast 21: 947-962.

6. Rother S, Burkert C, Brunger KM, Mayer A, Kieser A, et al. (2010) Nucleocytoplasmic shuttling of the La motif-containing protein Sro9 might link its nuclear and cytoplasmic functions. Rna 16: 1393-1401.

7. Mayer A, Lidschreiber M, Siebert M, Leike K, Soding J, et al. (2010) Uniform transitions of the general RNA polymerase II transcription complex. Nature structural & molecular biology 17: 1272-1278.

8. Strasser K, Masuda S, Mason P, Pfannstiel J, Oppizzi M, et al. (2002) TREX is a conserved complex coupling transcription with messenger RNA export. Nature 417: 304-308.

9. Mayer A, Heidemann M, Lidschreiber M, Schreieck A, Sun M, et al. (2012) CTD tyrosine phosphorylation impairs termination factor recruitment to RNA polymerase II. Science 336: 1723-1725.

10. Mayer A, Schreieck A, Lidschreiber M, Leike K, Martin DE, et al. (2012) The spt5 C-terminal region recruits yeast 3' RNA cleavage factor I. Molecular and cellular biology 32: 1321-1331.
